# Simultaneous Isotropic Omnidirectional Hypersensitive Strain Sensing and Deep Learning‐Assisted Direction Recognition in a Biomimetic Stretchable Device

**DOI:** 10.1002/adma.202420322

**Published:** 2025-01-31

**Authors:** Muzi Xu, Jiaqi Zhang, Chaoqun Dong, Chenyu Tang, Fangxin Hu, George G. Malliaras, Luigi G. Occhipinti

**Affiliations:** ^1^ Electrical Engineering Division Department of Engineering University of Cambridge Cambridge CB3 0FA UK; ^2^ Department of Electrical and Electronic Engineering University of Hong Kong Pokfulam Road Hong Kong SAR 999077 China

**Keywords:** biomimetic, deep learning, direction recognition, hypersensitive, isotropic omnidirectional sensing, stretchable strain sensor

## Abstract

Omnidirectional strain sensing and direction recognition ability are features of the human tactile sense, essential to address the intricate and dynamic requirements of real‐world applications. Most of the current strain sensors work by converting uniaxial strain into electrical signals, which restricts their use in environments with multiaxial strain. Here, the first device with simultaneous isotropic omnidirectional hypersensitive strain sensing and direction recognition (IOHSDR) capabilities is introduced. By mimicking the human fingers from three dimensions, the IOHSDR device realizes a novel heterogeneous substrate that incorporates the involute of a circle, resulting in isotropic behavior in the radial direction and anisotropic property in the involute direction for hypersensitive strain sensing. With the assistance of a deep learning‐based model, the IOHSDR device accomplishes an impressive accuracy of 99.58% in recognizing 360° stretching directions. Additionally, it exhibits superior performance in the typical properties of stretchable strain sensors, with a gauge factor of 634.12, an ultralow detection limit of 0.01%, and outstanding durability exceeding 15 000 cycles. The demonstration of radial artery pulse and throat vibration applications highlights the IOHSDR's unique characteristics of isotropic omnidirectional sensing and precise direction detection unleashing new classes of wearable health monitoring devices.

## Graphical abstract

Omnidirectional strain sensing is crucial in healthcare monitoring, human motion detection, and human‐machine interfaces. By mimicking the 3D structure of human fingers, this work introduces a novel heterogeneous substrate incorporating the involute of a circle which enables the device to achieve isotropic omnidirectional hypersensitive strain sensing and directional recognition simultaneously.

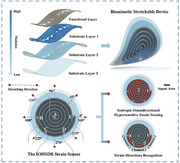

## 1 Introduction

Flexible and stretchable physical sensors possessing omnidirectional sensing capability are significant in response to the complex, variable, and dynamic real‐world scenarios in healthcare monitoring, human motion detection, and human‐machine interfaces.^[^ ^1^, ^2^, ^3^, ^4^, ^5^, ^6^, ^7^, ^8^, ^9^ ^]^ To quantify vibration and deformation stimuli, stretchable strain sensors play an essential role in the area of wearable electronics and electronic skins, with the advantages of high flexibility, simplicity, and conformability.^[^ ^10^, ^11^, ^12^, ^13^ ^]^ Remarkable achievements have been made in the development of stretchable strain sensors, focusing on enhancing their sensitivity, stretchability, durability, hysteresis, and detection limits by utilizing novel nanomaterials and micro/nanostructures.^[^ ^14^, ^15^, ^16^, ^17^ ^]^ Notably, outstanding sensitivity within a small strain range is required to enable the sensor to detect tiny biophysical signals such as pulse waves and throat vibrations.^[^ ^18^, ^19^, ^20^ ^]^ However, most stretchable strain sensors with excellent performance are constrained by the ability to transduce only uniaxial strain into electrical signals due to their intrinsic properties, such as the structure with a large length‐to‐diameter ratio and sensing materials with unidirectional distribution,^[^ ^21^, ^22^, ^23^, ^24^, ^25^, ^26^, ^27^ ^]^ hindering their application in a multiaxial strain environment. Consequently, there is an urgent need to develop more sophisticated strain sensor systems capable of effectively perceiving complex information that encompasses strains from various directions.

Recently, to detect more complex multiaxial strain conditions, omnidirectional strain sensing technology has been developed primarily via two strategies: single sensor and multi‐sensor systems. In the single sensor scheme, certain isotropic omnidirectional flexible strain sensors were devised using curved microgrooves arranged around a circle^[^ ^28^ ^]^ and incorporating chiral auxetic metamaterials into a substrate.^[^ ^29^ ^]^ While these sensors can detect strain from multiple directions with high sensitivity, they cannot determine the specific direction of the strain with a single sensor, and an additional sensor array is required. Previous attempts to achieve directional strain sensing are based on a multi‐sensor system approach typically involving two or three anisotropic strain sensors positioned at a specific angle and bespoke algorithms to calculate the intensity and direction of strain based on the signal difference between each sensor,^[^ ^30^, ^31^, ^32^ ^]^ which are fundamentally different from the single sensor approach proposed in this work (see Table ST1, Supporting Information). Additionally, due to the lack of isotropic property, the multi‐sensor system approach is more complicated when it comes to process a multi‐directional strain (i.e., the same strain applied in different directions). Technically, it is highly challenging to achieve both isotropic omnidirectional strain sensing and direction recognition within a single sensor, as the underlying principles are fundamentally opposite. Isotropic sensing requires a homogeneous platform to output identical responses, whereas direction recognition relies on signals' discrepancies. Thus, a feasible strategy to combine these two features is critically required for various practical applications, given the anticipated simplicity and efficiency of the sensor.

To address this significant issue, we turn our attention to biomimicry inspired by human fingers, renowned for their hypersensitive omnidirectional tactile perception capabilities. Based on the three dimensions mimicking, we successfully developed, to the best of our knowledge, the first single strain sensor that simultaneously integrates isotropic omnidirectional hypersensitive strain sensing and direction recognition, designated as the IOHSDR. This sensor consists of a three‐layer substrate with a gradient modulus and a functional layer. Leveraging the properties of the involute of a circle, the three‐layer heterogeneous substrate exhibits isotropy in each radial direction while demonstrating anisotropy in the involute direction. This unique substrate design effectively meets the seemingly contradictory requirements of isotropic sensing and direction recognition, resulting in the IOHSDR strain sensor's capability for dynamic switching between isotropic and anisotropic states. Furthermore, the employment of a deep learning‐based model achieves an exceptional direction classification accuracy of 99.58%.

## 2 Results and Discussions

### 2.1 IOHSDR Strain Sensor

The multidimensional biomimetic IOHSDR stretchable device imitates human fingers in three aspects: structure, surface morphology, and material. As mentioned, such biomimicry enables a single sensor to sense omnidirectional strains with isotropic hypersensitivity while simultaneously accurately recognizing directions.

**Figure 1:**
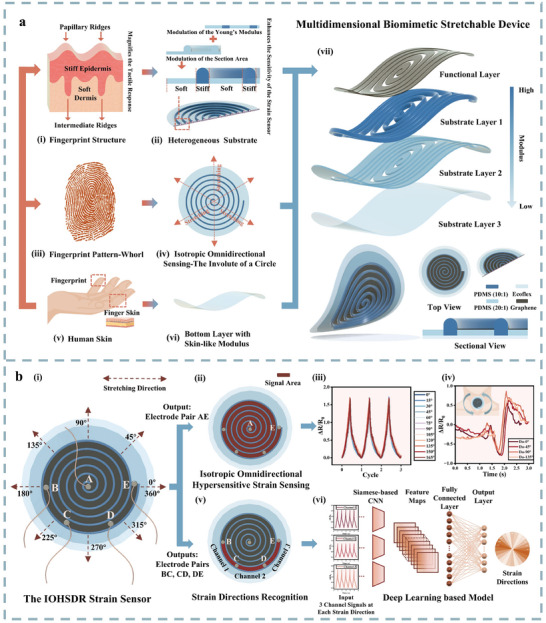
Schematic illustrations of the IOHSDR (isotropic omnidirectional hypersensitive sensing and direction recognition) strain sensor system. a) Schematic diagram of multidimensional biomimetic IOHSDR strain sensor design and structure, mimicking human fingers from three aspects: (i) fingerprint structure inspires (ii) heterogeneous substrate providing hypersensitivity; (iii) fingerprint whorl pattern inspires (iv) the involute of a circle as the ridges of substrate providing isotropic omnidirectional strain sensing; (v) soft human skin inspires (vi) skin‐like bottom layer for better strain transduction; (vii) the IOHSDR strain sensor structure consisting of a functional layer and a three‐layer substrate with the gradient of modulus. b) Schematic diagram of the IOHSDR strain sensor functions: (i) single IOHSDR strain sensor with stretchable silver electrodes A‐E; (ii‐iv) omnidirectional (360°) strains and throat vibrations detected from the electrode pair AE; (v‐vi) strain direction recognition via deep learning‐based model using three‐channel signals from the electrode pairs BC, CD and DE.

Specifically, fingerprints, as an indispensable part of the finger, play a vital role in tactile perception because of their unique interlock structure and modulus contrast (**Figure** **1**a(i)). The papillary ridges on the stiff epidermis and the mirrored intermediate ridges formed by the protrusion from the epidermis into the soft dermis function as numerous small mechanical levers to magnify the tactile response.^[^ ^33^, ^34^, ^35^ ^]^ Inspired by this, we utilize strain engineering to fabricate a heterogenous substrate that boots sensitivity through the integration of two modulation mechanisms (Figure 1a(ii)). Compared with a homogeneous substrate, the heterogeneous strain distribution of an elastomer substrate confers higher sensitivity to stretchable strain sensors.^[^ ^36^ ^]^ Among them, the modulation mechanism of Young's modulus^[^ ^37^ ^]^ and the section area^[^ ^38^ ^]^ have been investigated individually. For enhanced sensitivity, the reciprocal of Young's modulus or cross‐sectional area in a specific segment should exceed the average of these reciprocal values across the entire structure, allowing the local strain to surpass the total strain.^[^ ^36^ ^]^ Based on this principle, we simultaneously regulated Young's modulus and the section area of the substrate, mimicking the fingerprint structure to create a hypersensitive strain sensor with gauge factor 634.12.

Second, the fingerprint reaches the strongest amplification effect when strain is applied perpendicular to the papillary ridges.^[^ ^39^ ^]^ And the whorl‐shaped fingerprint (Figure 1a(iii)) ensures that no matter how the fingers move, certain ridges are always in an optimal position to generate the maximum response.^[^ ^40^, ^41^, ^42^ ^]^ Such patterns and performance provide an effective solution for isotropic omnidirectional sensing. To replicate this functionality, we adopted the involute of a circle as the ridge pattern of our sensor (Figure 1a (iv)). The involute of a circle can be described by unwinding a string from the circumference of the circle so that the distance between each loop is a constant determined by the perimeter of the base circle.^[^ ^43^ ^]^ By implementing this pattern on our heterogeneous substrate, the modified substrate provides a 360° continuous area from center to periphery for functional materials, and regardless of the strain's direction, the involute of a circle remains perpendicular to the strain, inducing the maximum response of the functional material to achieve isotropic omnidirectional sensing.

Moreover, to facilitate the adoption of the proposed strain sensor in wearable electronics, it is essential to minimize the mechanical mismatch between it and human skin, especially the modulus mismatch.^[^ ^44^ ^]^ The smaller the modulus mismatch, the better the conformability of the stretchable strain sensor on non‐zero Gaussian surfaces like human skin.^[^ ^45^ ^]^ To this purpose, we selected Ecoflex 00–10, a biocompatible material with skin like Young's modulus^[^ ^45^, ^46^ ^]^ as the bottom substrate layer of our strain sensor (Figure 1a (v‐vi)), achieving high fidelity of strain transduction for human health monitoring and motion detection.

Consequently, the IOHSDR strain sensor is developed, with its structure and materials composition illustrated in Figure 1a (vii). As the ridge of the sensor, Substrate‐Layer 1 (SL1) shapes as the involute of a circle featuring a high modulus. Substrate‐Layer 2 (SL2), formulated by cutting the projection of SL1 out of a thinner film with a medium modulus, forms a heterogeneous substrate when nested with SL1, contributing to hypersensitivity. Substrate‐Layer 3 (SL3), with a low modulus, acts as the bottom layer, in contact with the skin to transduce strain. Finally, a functional material fills the spaces between the loops on top of SL2, creating a continuous sensing area in the Functional Layer (FL). The fabrication process and dimensions of the IOHSDR strain sensor are detailed in Note S1 and Figure S1–S3 (Supporting Information). The fabricated IOHSDR device demonstrates consistent mechanical properties across an extended range of strain up to 100% the original length of the device, as evidenced by the force–strain curve in Figure S4 (Supporting Information). This performance benefits from the stable and robust interfacial bonding between different substrate layers and the functional layer (Note S2 and Figure S5, Supporting Information). With the addition of stretchable electrodes A, B, C, D, and E, this innovative stretchable device (photograph of the physical IOHSDR device in Figure S6, Supporting Information) can detect isotropic omnidirectional (360°) strains and throat vibrations, providing abundant information directly from the AE electrode pair (Figure 1b (i‐iv)). Meanwhile, strain direction is identified through a deep learning model with a Siamese‐based Convolutional Neural Network (CNN) as the backbone, utilizing three‐channel signals from electrode pairs BC, CD, and DE (Figure 1 b (v‐vi)).

**Figure 2:**
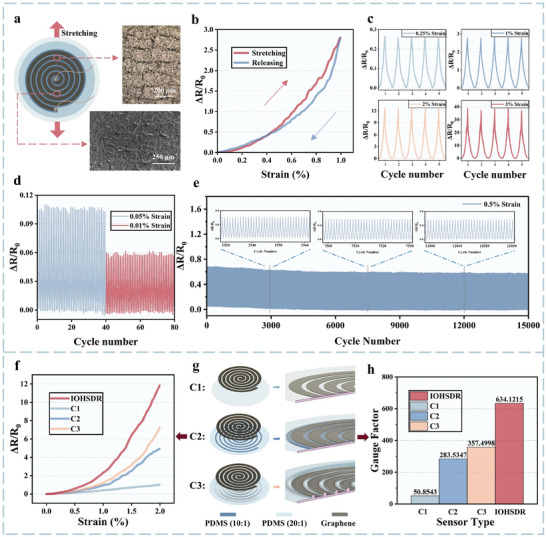
Characterization of the device. a) Microscope (above) and SEM (below) images of the microcracks of the graphene functional layer along the stretching direction, scale bar: 200 and 250 µm, respectively. b) Hysteresis of the relative resistance change during a stretching‐releasing cycle. c) Relative resistance change under 0.25%, 1.0%, 2.0%, and 3.0% cyclic strains. d) Detection limit stability test under 0.05% and 0.01% cyclic strains. e) Durability test by multicyclic stretching and releasing over 15 000 cycles under 0.5% strain. f) Comparison of relative resistance changes between the strain sensors IOHSDR, C1, C2, and C3. g) Structure and section view of C1, with an unmodulated substrate; C2, with a substrate modulated by Young's modulus; and C3, with a substrate modulated by cross‐sectional area. h) Comparison of gauge factors between the strain sensors IOHSDR, C1, C2, and C3.

Before demonstrating the two primary features of the IOHSDR strain sensor, we first investigated its fundamental characteristics and functioning mechanisms at the basis of the excellent performance results shown in **Figure** **2**. These results provide a solid foundation for its isotropic omnidirectional strain sensing and direction recognition capabilities. Founded upon a piezoresistive strain response mechanism, the IOHSDR strain sensor converts external vibrations and deformations into changes in electrical resistance, further enhanced by the novel heterogeneous substrate structure, in combination with graphene nanoplatelets, which enhance the piezoresistive properties due to their inherent defects^[^ ^47^ ^]^ and maintains a stable performance around the body temperature range (Figure S7, Supporting Information). Preparing the functional ink via a simple high‐pressure homogenization (HPH) method (Figure S8, Supporting Information), a uniform graphene nanoplatelets ink (characterized in Figure S9, Supporting Information) is sprayed onto the surface of the SL2. Following oxygen plasma treatment, the hydrophobic silicone rubber is modified by introducing silanol (Si−OH) terminal groups, allowing strong chemical bonding between the functional material and the substrate, thereby preventing delamination during the repeated strain in the use‐case application.^[^ ^48^, ^49^ ^]^ The enhanced interfacial bonding resulting from this modification (Figure S10, Supporting Information) is instrumental to ensure the durability of the sensor in spite of the mechanical mismatch between the two layers. Upon stretching, the microcracks, identified as an effective mechanism for boosting the sensitivity of strain sensors,^[^ ^50^, ^51^ ^]^ form along the strain direction between each loop (Figure 2a). The involute of a circle structure facilitates the continuous spiral sensing area to accumulate resistance changes from each revolution along the stretching direction, significantly improving the sensitivity. As a resistive strain sensor, the performance of the IOHSDR sensor is evaluated by analyzing the variations in relative resistance. For tiny deformation and vibration detection, it is noted that our sensor displays a relatively low hysteresis (Figure 2b), indicating robust interfacial bonding between nanomaterials and polymer substrates, which ensures good dynamic strain sensing performance^[^ ^52^, ^53^ ^]^ and establishes a strong foundation for reliable signal acquisition. Stable stretching‐releasing responses under varying strain levels are critical for subsequent direction analysis, particularly within small strain ranges. The IOHSDR strain sensor exhibits excellent repeatability across different strain conditions (Figure 2c). In addition, the smallest quantity that a strain sensor can measure with a specified precision determines the applicable scenarios, because a large detection limit may lead to the omission of critical information during perception and extraction, greatly hindering its implementation in human health monitoring.^[^ ^54^ ^]^ Benefiting from the amplification effect of our innovative involute heterogeneous substrate, the IOHSDR strain sensor demonstrates an ultralow detection limit of ≈0.01% strain (Figure 2d), satisfying the requirement for precise perception. Moreover, our proposed sensor can withstand over 15 000 cycles of stretching and releasing without degradation of electrical performance and mechanical integrity (Figure 2e), outperforming most reported sensors. This extraordinary durability equips the IOHSDR strain sensor with reliable and enduring omnidirectional sensing and direction recognition functions.

Combining two modulation mechanisms, our heterogeneous substrate introduces a regulation effect as the basis for sensitivity enhancement, which was analyzed through comparative experiments with strain sensors featuring substrates modulated by a single property or without modulation. The comparative experimental group includes three strain sensors: C1, with an unmodulated substrate; C2, with a substrate modulated by Young's modulus; and C3, with a substrate modulated by cross‐sectional area. When a strain was applied to sensors C1‐C3 and the IOHSDR strain sensor, each exhibited distinct strain distributions due to the different regulation effects of their substrates. Finite element analysis (FEA, Figure S11, Supporting Information) reveals that the IOHSDR strain sensor achieves the highest local strain compared to sensors C1 through C3, in the following order: IOHSDR > C3 > C2 > C1. And this strain distribution result is reflected in the sensitivity characterization (Figure 2f–h). Notably, the IOHSDR strain sensor displayed the highest resistance response, with a gauge factor (GF) of up to 634.12 (where GF = (ΔR/R₀)/ɛ, with ΔR/R₀ representing the relative change in resistance and ɛ the strain). Consistent with the FEA results, the IOHSDR sensor achieves a 12‐fold sensitivity enhancement over the unmodulated substrate. The sensitivities of C2 and C3, each employing substrates modulated by a single property, are approximately half that of the IOHSDR sensor. These findings highlight the effectiveness of the dual modulation mechanisms in achieving hypersensitivity, as demonstrated by both FEA and gauge factor analysis in the comparative experiments.

### 2.2 Isotropic Omnidirectional Hypersensitive Strain Sensing

Isotropic omnidirectional hypersensitive strain sensing refers to the sensor's ability to reliably detect even minute strains from all 360° directions with uniform responsiveness. Owing to the superior fundamental properties discussed above, the IOHSDR strain sensor outputs consistent performance regardless of the strain direction in both characterization and application tests. Prior to analyzing the results of omnidirectional sensing, a mathematical explanation of the involute of a circle is provided to elucidate the mechanisms underlying the realization of both omnidirectional and magnified strain sensing. To create a continuous sensing area that ensures a uniform response across 360° while amplifying signals, we select the involute of a circle as the structural design for the substrate ridges rather than the Archimedean spiral, which features non‐parallel tangents for each loop. The involute of a circle is generated by tracing the trajectory of a point on a taut string as it unwinds from the perimeter of the circle. When this point moves away from the base circle, the distance it travels is directly proportional to the unwinding angle, resulting in uniformly spaced loops. Based on its generation method, all the normal of the involute of a circle are tangent to the base circle, and the tangents of each loop of the involute are parallel. In other words, the set of involutes and the corresponding tangents to the base circle collectively constitute an orthogonal coordinate system. Such characteristics endow the IOHSDR strain sensor with isotropic omnidirectional sensing capability.

**Figure 3:**
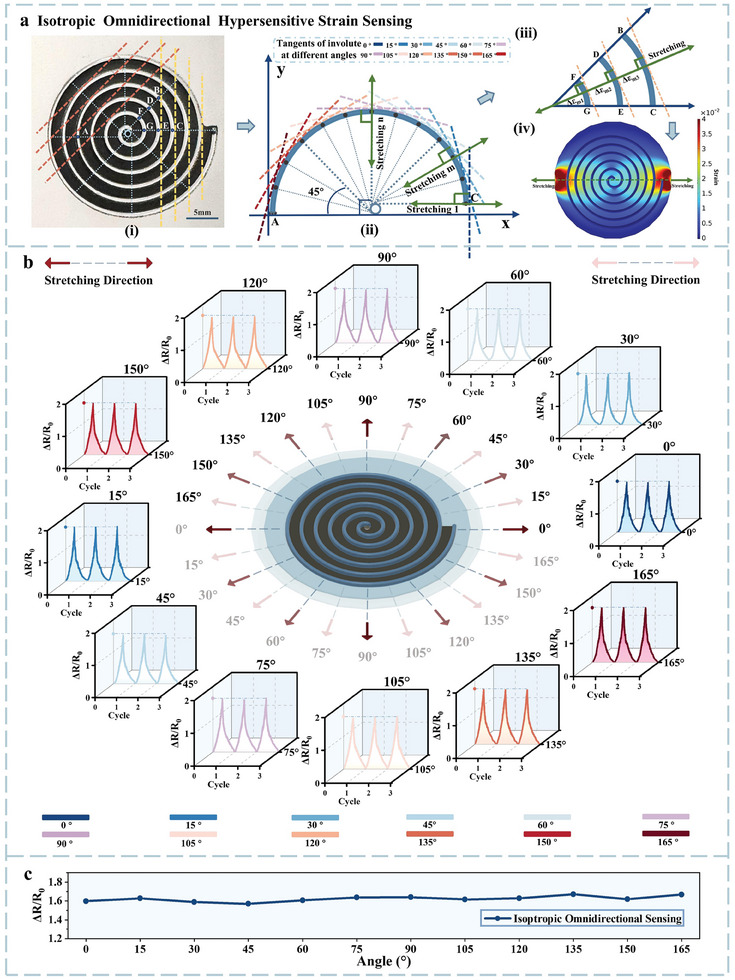
Isotropic omnidirectional hypersensitive strain sensing of the IOHSDR strain sensor. a) Mechanism of isotropic omnidirectional strain sensing: (i) top view physical photo of the IOHSDR strain sensor; (ii) mathematical analysis of isotropic omnidirectional strain sensing: regardless of the stretching directions, there is always an involute perpendicular to it to induce the maximum strain responses in the substrate; (iii) mathematical analysis of enhanced strain sensing: due to the parallel tangents of each involute loop, the stretching results in maximum strain occurring across all loops; (iv) FEA result of 0° stretching of the IOHSDR strain sensor. b) Isotropic omnidirectional (360°) strain sensing result of the IOHSDR sensor; strain of equal magnitude was applied at 12 angles spaced at 15° intervals, spanning a full 360° range. c) Relative resistance change curve for signal intensity across the 12 stretching directions.

**Figure** **3**a (i) presents the physical photo of the completed IOHSDR strain sensor. It is intuitive that the distances between each loop are equal, and the tangents for each loop are parallel. When the stretching direction is perpendicular to the involute, the substrate generates the maximum strain response. Given the intrinsic properties of the involute, regardless of the direction the stretching is implemented, there will always be an involute perpendicular to it. For instance, within the arc segment AC (180°) shown in Figure 2a (ii), stretching applied at 15° intervals from 0° to 180° can induce maximum strain responses in the substrate. In addition, due to the parallel tangents across each loop of the involute, the stretching results in maximum strain occurring consistently across all loops (Figure 2a (iii‐iv)). This means that each loop produces the maximum resistance response, which can be accumulated along the continuous conductive path from the center to the periphery. As a result, the IOHSDR strain sensor can detect deformations isotropically across a full 360° range.

Figure 3b describes the isotropic omnidirectional strain sensing results of the IOHSDR sensor, using the alternative strain testing setup depicted in Note S4 and Figures S12–S14 (Supporting Information). In this measurement, strain of equal magnitude was applied at 12 angles spaced at 15° intervals, spanning a full 360° range. Based on the diagrams illustrating the relative change in resistance for each tested angle, the IOHSDR strain sensor produces stable and repeatable output signals throughout the stretching‐releasing cycles and exhibits consistency in both signal pattern and magnitude for omnidirectional sensing. Figure 3c summarizes the signal intensity across the 12 stretching directions, offering a more comprehensive view of the sensor's isotropic omnidirectional sensing capability. The nearly linear curve composed of the 12 data points, with a standard deviation of 0.0288, indicates the sensor's exceptional performance in isotropic omnidirectional sensing, which aligns with the results obtained from the Finite Element Analysis (FEA) (Figure S15, Supporting Information).

**Figure 4:**
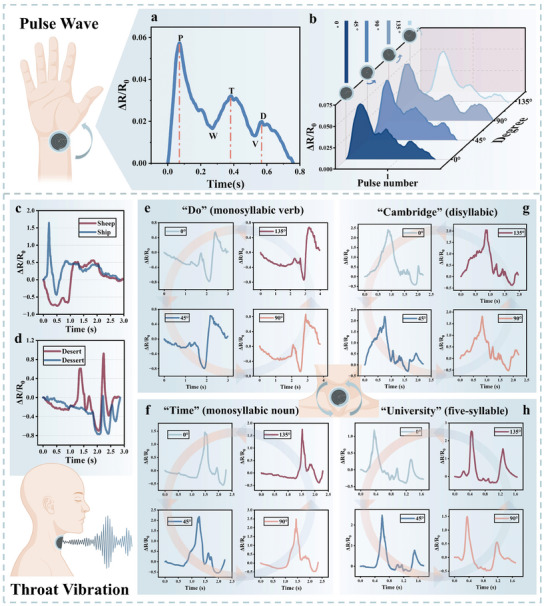
Demonstration of pulse wave monitoring and throat vibration detection based on IOHSDR strain sensor. a) The pulse waveform of the radial artery collected by the IOHSDR strain sensor, displaying detailed information including advanced wave (P), reflected notch (W), reflected wave (T), dicrotic notch (V) and dicrotic wave (D). b) Four pulse waveforms obtained by the IOHSDR sensor attached to the wrist at angles from 0° to 180° in 45° intervals. c,d) The throat vibration signals of word pairs: “Sheep” and “Ship”, and “Desert” and “Dessert”. e–h) Throat vibration signals of “Do” (monosyllabic verb), “Time” (monosyllabic noun), “Cambridge” (disyllabic), and “University” (five‐syllable) were obtained by altering the sensor's attachment angles on the throat skin, from 0° to 180° at 45° intervals.

To illustrate some of the benefits of isotropic strain sensing, the IOHSDR strain sensor is further tested in human health monitoring applications, specifically for detecting pulse waves and throat vibrations, both requiring high sensitivity. Pulse wave, as the most essential clinical indicator, plays an indispensable role in the early diagnosis of cardiovascular diseases.^[^ ^55^ ^]^ Typical arterial pulse wave features several peaks and notches, such as advancing wave, reflected wave, and dicrotic wave, relating to the heartbeat and blood pressure.^[^ ^56^ ^]^ However, conventional devices lack the high sensitivity and adequate conformability to capture detailed information from pulse waves. To facilitate in‐depth analysis of pulse waves for cardiovascular health, the IOHSDR strain sensor is conveniently adopted to provide highly precise pulse signals with abundant features content. **Figure** **4**a shows the pulse waveform of the radial artery collected by our novel sensor, revealing detailed components such as the advancing wave (P), reflected notch (W), reflected wave (T), dicrotic notch (V), and dicrotic wave (D). Based on this, Figure 4b presents four pulse waveforms recorded with the IOHSDR sensor attached to the wrist at angles from 0° to 180° in 45° intervals. Benefiting from its remarkable isotropic omnidirectional hypersensing properties, the sensor consistently detects waveforms that include the P, W, T, V, and D waves, regardless of its orientation.

The silent communication recognition system is important for those who cannot rely on physical voice signals, particularly for people suffering from speech and hearing impairments.^[^ ^18^ ^]^ Based on the fact that speaking different words corresponds to different degrees of stretching or shrinking strains of throat muscle,^[^ ^57^ ^]^ sensing strain and vibration generated on the throat skin surface offers a feasible solution.^[^ ^58^ ^]^ To accurately distinguish between different words, a strain sensor must possess hypersensitivity to extract rich features embedded in the original biosignals. The proposed IOHSDR strain sensor effectively discerns the fundamental signal characteristics, even between words with highly similar pronunciations, as illustrated by word pairs: “Sheep” and “Ship”, and “Desert” and “Dessert” (Figure 4c,d). Furthermore, the sensor's isotropic omnidirectional sensing capability is validated by altering the sensor's attachment angles on the throat skin. Words from different categories and syllable counts, such as “Do” (monosyllabic verb), “Time” (monosyllabic noun), “Cambridge” (disyllabic), and “University” (five‐syllable), were used to evaluate the performance. Four groups of distinct omnidirectional speaking vibration signals are presented in Figure 4e–h. Despite both being monosyllabic, “Do” and “Time” exhibit entirely different response signals. For “Cambridge” and “University”, the increased syllable count from two to five does not compromise the stability of the isotropic omnidirectional response. This implies that the sensor can detect subtle differences between similar words while maintaining consistent and stable omnidirectional sensing. Overall, the IOHSDR sensor's isotropic omnidirectional hypersensitive strain sensing capability is comprehensively demonstrated through test characterization and health monitoring applications.

### 2.3 Deep Learning‐Assisted Direction Recognition

**Figure 5:**
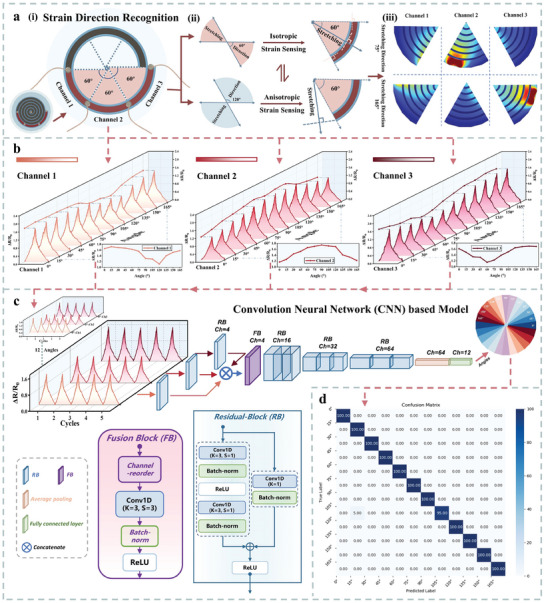
Deep learning‐assisted direction recognition. a) Mechanism of direction recognition: (i) three channels for 360° strains direction recognition; (ii) analysis of the channel that dynamically switches between isotropic and anisotropic states; (iii) FEA results for three channels under the stretching from 75° and 165°. b) Relative resistance change and magnitude trends curves of Channel 1, Channel 2, and Channel 3 during the stretching from 0° to 180° at 15° intervals. c) The structure of the Convolution Neural Network (CNN) based model for IOHSDR strain sensor recognizing the stretching direction. d) Confusion matrix for the classification of 12 stretching directions from 0° to 180° at 15° intervals.

Leveraging its unique structural design, the IOHSDR strain sensor not only achieves exceptional isotropic omnidirectional sensing but also enables strain direction recognition with remarkable accuracy. In the IOHSDR strain sensor, a continuous sensing area follows the pattern of the involute of a circle extending from the center to the periphery. This structure achieves isotropy in the radial direction and anisotropy in the involute direction, creating discrepancies between signals within specific sensing areas. In light of this, a segment of the involute sensing area is selected to distinguish stretching directions. Due to the symmetric nature of stretching, this segment only needs to encompass a 180° strain response area to facilitate omnidirectional recognition, as shown in **Figure** **5**a (i). The identification of strain directions is accomplished by analyzing several different signals, so the selected segment with additional electrodes is divided into three equal channels based on the angle, to ensure 360° recognition while simplifying direction analysis with the minimum number of channels. To investigate how these three channels distinguish strain directions, we use Channel 3 as an example to illustrate its mechanism in Figure 5a (ii). When stretching occurs within the range of Channel 3 (60°), there is always an involute perpendicular to it to induce the maximum strain responses, resulting in isotropic strain sensing. In contrast, when stretching originates from the directions outside the range of Channel 3, the angle between the stretching direction and the involute of Channel 3 becomes variable. This variability indicates that the strain responses from this channel are contingent upon the stretching direction, leading to anisotropic strain sensing. Therefore, Channel 3 can dynamically switch between isotropic and anisotropic states based on the strain direction, and signals from such three channels provide information about stretching directions. According to the FEA results presented in Figure 5a (iii), it is clear that the three channels yield distinct strain responses when subjected to stretching from the 75° and 165° directions, respectively. For example, stretching from 75° induces the largest strain on Channel 2, while Channel 3 experiences the smallest strain. Consequently, collected from three channels, Figure 5b presents the results of relative resistance changes observed during the stretching from 0° to 180° at 15° intervals. Each channel shows different signal trends across all tested directions, with Channel 1 and Channel 3 exhibiting opposite trends and Channel 2 displaying a symmetrical peak. The comparison of the three‐channel signals, based on data collected from the IOHSDR strain sensor and finite element analysis (FEA) simulations, is provided in Figures S16 and S17 of the Supporting Information.

Taking into account the direction‐related signals from the 3 channels, we utilize the superior features extraction and powerful fusion capabilities of data‐driven deep learning methods to enable the IOHSDR sensor to achieve high accuracy direction recognition. For the backbone selection of our model, it is important to note that though Transformer^[^ ^59^ ^]^ networks, often considered high‐capacity architectures, usually need large training datasets in order to ensure accurate performance. Conversely, convolutional neural networks (CNNs) are more suitable for small‐scale datasets. Additionally, the inductive bias of convolution operation, including the local receptive field, the features hierarchy, and translation invariance, are powerful characteristics, sufficient to extract detailed features from our sensor data with limited length. As illustrated in Figure 5c, we adopt a Residual Neural Network (ResNet)^[^ ^60^ ^]^ with 1D convolution as our backbone. Considering the unique characteristics of the three direction‐related channels, we propose a triple‐branch architecture to separately extract the distinct features from each channel. Then, inspired by Polar Eyeball Shape Net (PESNet),^[^ ^61^ ^]^ a channel‐wise fusion block is employed to fuse the extracted features from the triple‐branch. Specifically, this block first rearranges the order of channels in concatenated feature volumes according to the directional correlations, then uses 1D convolution with (3 × 3) kernel and (3 × 1) stride to combine the features from the same channel of triple‐branch. Finally, after 3 sets of 1D Residual Blocks, the final fully connected layer produces the class prediction output, i.e., the direction of strain. For this strain direction classification task, we collected 3600 sets of direction‐related resistance data directly from three channels, with the detailed dataset information provided in Figures S18 and S19 (Supporting Information). After pre‐processing the raw datasets, 70% were allocated for training the model, and 20% and 10% were used for validation and testing, respectively. During the training process, we use the cross entropy loss to constrain our model for strain direction recognition. As shown in Figure S20 (Supporting Information), both the train and validation loss curves decline promptly and reach convergence within tens of epochs, which demonstrates the stability and efficiency of our model. As a result, our proposed model achieved a classification accuracy of 99.58% for the 12 angles from 0° to 180°, and the corresponding confusion matrix is displayed in Figure 5d. The proposed method effectively distinguishes the directional pattern to classify the right stretching direction based on the signals from 3 channels, endowing the IOHSDR strain sensor capability for accurate direction recognition across a full 360° range.

## 3 Conclusion

Herein, we proposed a multidimensional biomimetic stretchable device that simultaneously enables isotropic omnidirectional hypersensitive strain sensing and direction recognition. By mimicking the human finger from three anatomical characteristics, the IOHSDR device fulfills the requirements for both functionalities. Our device demonstrates isotropic behavior in the radial direction while exhibiting anisotropic properties in the involute direction for strain sensing. This unique performance is achieved through a uniform continuous active area that extends from the center to the periphery, which is facilitated by the innovative incorporation of the involute pattern and the unique composition of the functional and substrate layers. With the assistance of a deep learning‐based model, the IOHSDR achieves a remarkable accuracy of 99.58% in recognizing stretching directions. Furthermore, it exhibits outstanding performance in fundamental properties of stretchable strain devices, including a gauge factor of 634.12, an ultralow detection limit of 0.01%, and superior durability exceeding 15 000 cycles. These characteristics enable a wide range of applications in precise vibration and deformation detection, such as monitoring radial artery pulse and throat vibration associated with speech. Given the foundational aspects of the proposed design and device architecture, we see a potential for future works exploring the use of other materials in the functional layer with similar properties to graphene, such as MXenes, another class of 2D materials that exhibit innate high conductivity^[^ ^62^ ^]^ and strain sensing ability,^[^ ^63^ ^]^ after solving their initial weaknesses in terms of mechanical fragility and vulnerability to oxidation.^[^ ^64^ ^]^ The design principles can be adapted for various scenarios by modifying materials and dimensions to meet specific requirements. Thus, the IOHSDR stretchable device will open the door for complex, adaptive, and dynamic strain sensing in future healthcare monitoring, human motion detection, and human‐machine interfaces.

## 4 Experimental Section

### Fabrication of the Heterogenous Substrate

The heterogeneous substrate with a gradient modulus was fabricated using 3D‐printed molds and the spin coating method. The modulus of the three layers was controlled by adjusting the mass ratios of the prepolymer and curing agent. Specifically, polydimethylsiloxane (PDMS) with weight ratios of 10:1 and 20:1 (Dowsil SYLGARD 184 Silicone Elastomer Kit) was used to prepare Substrate Layer 1 (SL1) and Substrate Layer 2 (SL2), respectively. For Substrate Layer 3 (SL3), Ecoflex 00–10 was used in a 1:1 weight ratio. These three layers were fabricated sequentially at 80 °C, in descending order of modulus, as detailed in Note S1 and Figures S1 and S2 (Supporting Information).

### Fabrication of the IOHSDR Strain Sensor

The functional water‐based graphene nanoplatelet ink (50 g L^−1^) was prepared using a High Pressure Homogenizer (PSI‐40), with sodium deoxycholate (SDC) (≥97%) and sodium carboxymethyl cellulose (CMC‐Na) serving as the surfactant and binder. Before spray coating the graphene onto the substrate, O₂ plasma treatment (RIE‐Femto) was applied for 2 min to introduce silanol (Si−OH) terminal groups on the surface of PDMS SL2. The ink was then sprayed at 20 psi pressure for 2 min. Finally, stretchable silver electrodes were fabricated at 80 °C.

### Characterization and Demonstration of the Device

The morphology of the IOHSDR strain sensor was characterized using SEM (Magellan 400), with a gold layer sputter deposited onto the samples prior to testing. The strain sensing performance of the device was evaluated by the INSTRON universal tensile testing system, which applied predefined strain based on two testing methods outlined in Note S4 and Figures S13 and S14 (Supporting Information). The corresponding resistance response during cyclic stretching‐releasing was measured using PalmSens Multichannel Potentiostats with a sampling rate of 500 Hz. Additionally, the mechanical property of the sensor was assessed with the Hounsfield Universal Testing Machine (max 1 kN), which performed tensile force–strain testing over a strain ranges up to 100% of the sensor's original length. The omnidirectional pulse waveform of the radial artery and throat vibration biosignals were detected by the IOHSDR strain sensor, which was attached to the wrist and neck skin surfaces from 0° to 180° at 45° intervals, respectively. The electrical signals generated by these skin deformations were then recorded by PalmSens Potentiostats.

### Siamese‐Based Convolutional Neural Network (CNN) Deep Learning Approach

A total of 3600 sets of direction‐related resistance data were collected from three channels of the IOHSDR strain sensor, with each direction consisting of 100 samples per channel. The raw datasets were scaled to a range between 0 and 1 using Min–Max normalization. 70% of the data was allocated for training the model, while 20% and 10% were used for validation and testing, respectively. In the training and inference process, the standard cross entropy loss function was used to constrain the model. The model was implemented using the PyTorch framework, and the Adam optimizer with a learning rate of 2.5 × 10^−4^ was employed to train the model. The novel triple‐branch architecture separately extracted distinct features from each channel and achieved 99.58% accuracy in strain direction recognition after feature fusion based on the channel‐wise fusion block.

### Ethics Approval and Human Research Participants

The experiment involving human participants was approved by the Ethics Committee of the Department of Engineering at the University of Cambridge. All participants were provided with a Participant Information Sheet and asked to complete and sign a Participant Consent Form prior to their participation in the experiment.

### Statistical Analysis

All datasets for direction recognition were preprocessed using Min–Max normalization. The standard deviation (SD) of the magnitude of relative resistance change was calculated to assess the performance in isotropic omnidirectional sensing. No statistical tests were conducted in this study.

## Conflict of Interest

The authors declare no conflict of interest.

## Supporting information



Supporting Information

Supplemental Video 1

Supplemental Video 2

## Data Availability

The data and code supporting this study are available from the University of Cambridge repository, http://doi.org/10.17863/CAM.112973.
