# Supplementary material for: Simultaneous Isotropic Omnidirectional Hypersensitive Strain Sensing and Deep Learning‐Assisted Direction Recognition in a Biomimetic Stretchable Device
Source: Adv Mater. 2025 Jan 31;37(17):2420322. doi: 10.1002/adma.202420322 (PMC12038543; doi:10.1002/adma.202420322)
Supplement: Supplementary file 1 — Supporting Information [file ADMA-37-2420322-s002.docx]

Supporting Information

**Simultaneous Isotropic Omnidirectional Hypersensitive Strain Sensing and Deep Learning-Assisted Direction Recognition in a Biomimetic Stretchable Device**

**Authors**

*Muzi Xu^1^, Jiaqi Zhang^2^, Chaoqun Dong^1^, Chenyu Tang^1^, Fangxin Hu^1^, George G. Malliaras^1^, Luigi G. Occhipinti^*1^*

**Affiliations**

^1^Electrical Engineering Division, Department of Engineering, University of Cambridge, Cambridge, UK

^2^Department of Electrical and Electronic Engineering, University of Hong Kong, Pokfulam Road, Hong Kong SAR, China

^*^Correspondence to: [lgo23@cam.ac.uk](mailto:lgo23@cam.ac.uk)

**Content: Pg.**

Supplementary Notes 1-4 2

Supplementary Figures S1-S20 7

Supplementary Table ST1 27

References 28

**Supplementary Note 1:** **Fabrication of the IOHSDR Strain Sensor**

**Material:**

Dowsil SYLGARD™ 184 Silicone Elastomer Kit (prepolymer and curing agent) was purchased from Conro Electronics Ltd for the substrate layers 1 and 2. Ecoflex™ 00-10 (Part A and Part B) was purchased from Bentley Advanced Materials for the substrate layer 3. TIMREX KS 25 Graphite (synthetic graphite with a particle size of 25μm) was purchased from IMERYS. The sodium deoxycholate (SDC) (≥97%) and sodium carboxymethyl cellulose (CMC-Na: average molecular weight of 700,000) as the surfactant and the binder for ink preparation, were both obtained from SIGMA-ALDRICH. 3D printed Mould 1&2 and Masks for the substrate fabrication were purchased from PROTO LABS LTD. Stretchable printable silver paste DM-SIP-2006 was purchased from DYCOTEC MATERIALS LTD for electrodes.

**Fabrication of the Heterogeneous Substrate:**

The fabrication of the heterogeneous substrate with three layers follows below steps, using Mould 1&2 and Masks shaped like the involute of a circle.

*Substrate Layer 1:*

1. Spray a releasing agent on the surfaces of Moulds 1 & 2, and allow it to dry at room temperature.
2. Combine Mould 1 and 2, then pour PDMS 1 (Prepolymer : Curing agent = 10:1) onto the involute area.
3. Use a blade to remove excess PDMS 1, degas it in a vacuum oven, and cure in the oven for 2 hours at 80℃.

*Substrate Layer 2:*

1. Use a tweezer to remove the Mould 2, pour PDMS 2 (Prepolymer : Curing agent=20:1) onto Mould 1, degas it in a vacuum oven, and cure in the oven for 45 min at 80℃.

*Substrate Layer 3:*

1. Prepare Ecoflex 00-10 by mixing Part A and Part B in a 1:1 ratio, then degas it in a vacuum oven.
2. Spin-coat Ecoflex 00-10 on top of Mould 1 at 500 rpm for 180s (thickness ~ 150μm), then cure in the oven for 1 hour at 80℃.
3. Peel the three-layer substrate off Mould 1.

**Fabrication of Functional Graphene Nanoplatelets Ink:**

The functional graphene nanoplatelets ink is prepared by High Pressure Homogenizer (HPH), following the fabrication method we previously published.^[1]^

1. Dissolve SDC in deionized (DI) water (5 g/L) to prevent filler aggregation through electrostatic repulsion.
2. Add TIMREX KS 25 graphite flakes to the SDC solution (50 g/L) and mix with a dissolver at 500 rpm for 30 minutes.
3. Exfoliate the mixture using HPH (PSI-40) with a dual-slot deagglomeration chamber (D200D: 200 µm), operating at a pressure of 700 bar for 70 exfoliation cycles.
4. Add CMC-Na as a binder to the graphene dispersion (5 g/l) to stabilize the flakes and control the ink’s viscosity, and stir the prepared ink for 3 hours at room temperature to fully dissolve CMC-Na.

**Fabrication of the IOHSDR Strain Sensor**

1. Apply O_2_ Plasma treatment to introduce silanol (Si−OH) terminal groups on PDMS substrate layer 2.
2. Position the Masks over the substrate on a hotplate at 100°C, then spray 50 g/L graphene ink at 20 psi pressure, with a spray height of 23 cm and a spray duration of 2 minutes.
3. Fabricate electrodes using stretchable silver paste (cure in an oven at 80℃ for 40 minutes) and copper tape.

**Supplementary Note 2:** **Interfacial Bonding between Different Materials**

Our device consists of a three-layer substrate and a functional layer. The interfacial bonding between PDMS having different modulus, PDMS and Ecoflex, and PDMS and Graphene has been extensively studied.^[2–6]^ To enhance the interfacial bonding between these materials, we employed surface treatment techniques and adjusted the curing times during the fabrication process. By introducing silanol (Si−OH) terminal groups through oxygen plasma treatment, the Substrate Layer 1 (high-modulus PDMS) forms strong bonds with Substrate Layer 2 (low-modulus PDMS) and the Functional Layer (graphene nanoplatelets), effectively reinforcing interfacial adhesion.^[2,3]^ Additionally, as indicated by prior studies,^[4,5]^ PDMS exhibits stronger interfacial bonding with Ecoflex when it is partially cured. Therefore, we spin-coated Substrate Layer 3 (Ecoflex) onto Substrate Layer 2 (low-modulus PDMS) before it was fully cured.

The SEM images of the surface and cross-sectional views (Figure S5, Supporting Information) confirm that the different parts of our sensor are well-bonded. To further validate the structural stability, we performed mechanical characterization, as shown in Figure S4, Supporting Information. The stable force-strain curve provides evidence of the strong interfacial bonding among Substrate Layers 1–3 during tensile testing. Finally, cross-sectional SEM images illustrating the interface between the functional graphene layer and the substrate layer, both in unstretched and stretched states (Figure S10, Supporting Information), provide further evidence of robust interfacial bonding. These results highlight the effectiveness of the interfacial bonding and explain the exceptional stability and durability of our sensor.

**Supplementary Note 3:** **Finite Element Analysis (FEA) Simulation**

The commercial FEA software COMSOL Multiphysics 6.2 was employed to analyse the strain distribution of the substrates for IOHSDR, C1, C2, and C3 strain sensors. Since Polydimethylsiloxane (PDMS) behaves as a linear elastic material under strains up to 20%, the linear elastic model was adopted to simulate the PDMS substrate under small strain conditions.^[7,8]^ The Young’s modulus for PDMS 1 (Prepolymer : Curing agent = 10:1) and PDMS 2 (Prepolymer : Curing agent = 20:1) were set to 1.75 MPa and 0.83 MPa,^[9]^ respectively.

**Supplementary Note 4:** **Strain Testing Methods**

We adopted two testing methods to characterize the strain response of our device. Method 1 is a conventional tensile strain testing approach (Figure S13 (a), (c), (e), Supporting Information), and it was used to apply plane stretching to the sensor for characterizing all fundamental device properties as shown in Figure 2.

After confirming the isotropic omnidirectional performance of our sensor using Method 1, we employed the equivalent alternative Method 2 (Figure S13 (b), (d), (f), Supporting Information) to collect a large volume of isotropic omnidirectional strain signals. To evaluate the impact of the two testing methods in the corresponding strain distribution, we conducted a comparative analysis of the experimental setups and derived a finite element model of the strain distribution (Figure S13 (e), (f), Supporting Information), confirming the consistency of the results obtained.

To further explain this result, we analysed in detail the working mechanism of Method 2 by dividing the sensor into three regions (Figure S14 (a), (b), Supporting Information), where Regions 1 and 3 experience plane stretching, and Region 2 undergoes bending. As shown in Figure S14 (c), Supporting Information we could derive the corresponding plane stretching strain percentage in the Regions 1 and 3 of the sensor, using Method 2. Due to the limited area affected by bending, the impact of bending on the sensor's tensile strain response can be considered negligible. Therefore, this approach allows for more efficient equivalent testing of isotropic omnidirectional performance, especially when testing at numerous angles and requiring large datasets.

**
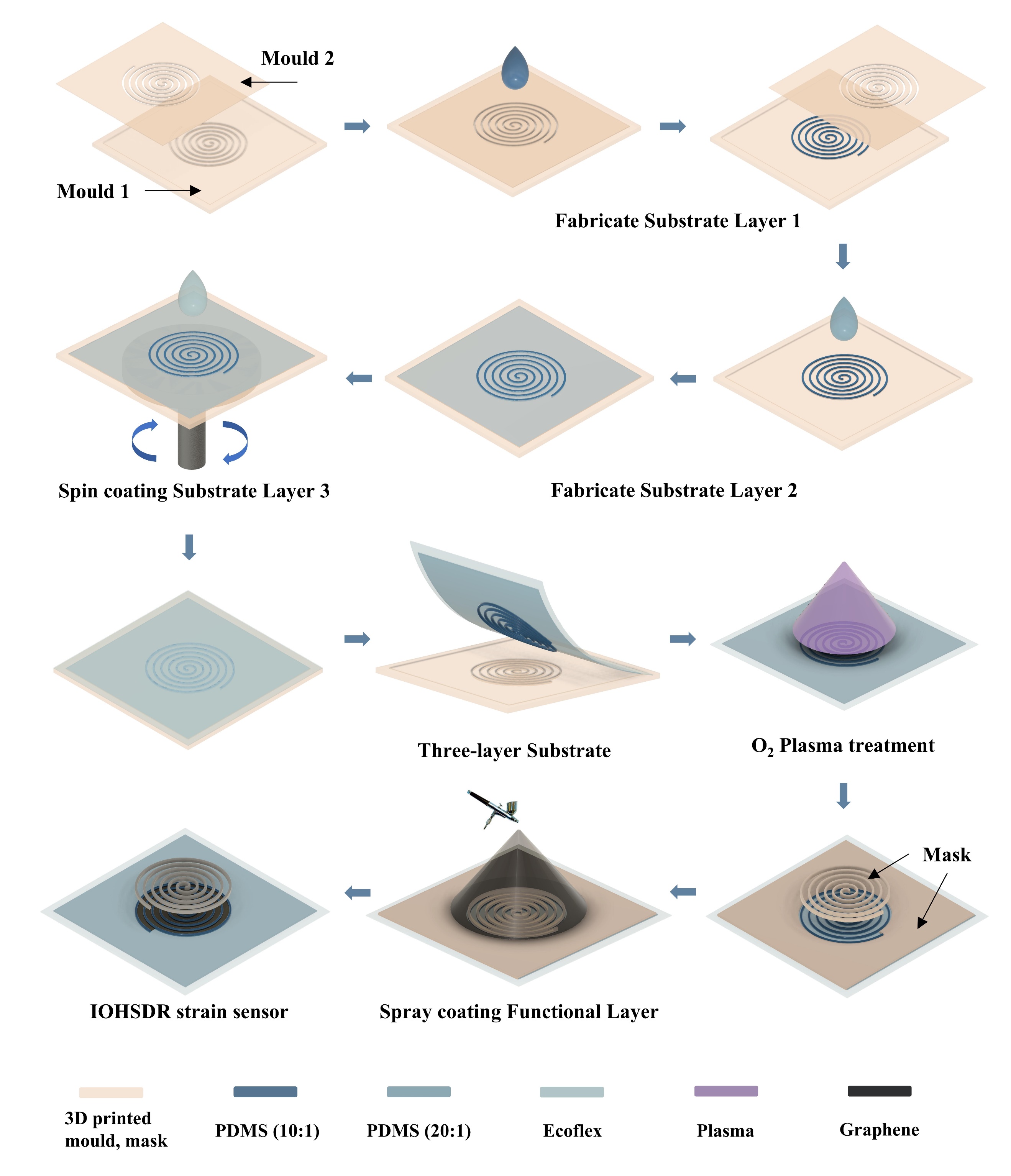
**

**Figure S1.** Schematic of the fabrication of the IOHSDR strain sensor. The detailed steps are illustrated in the Supplementary Note1.

**
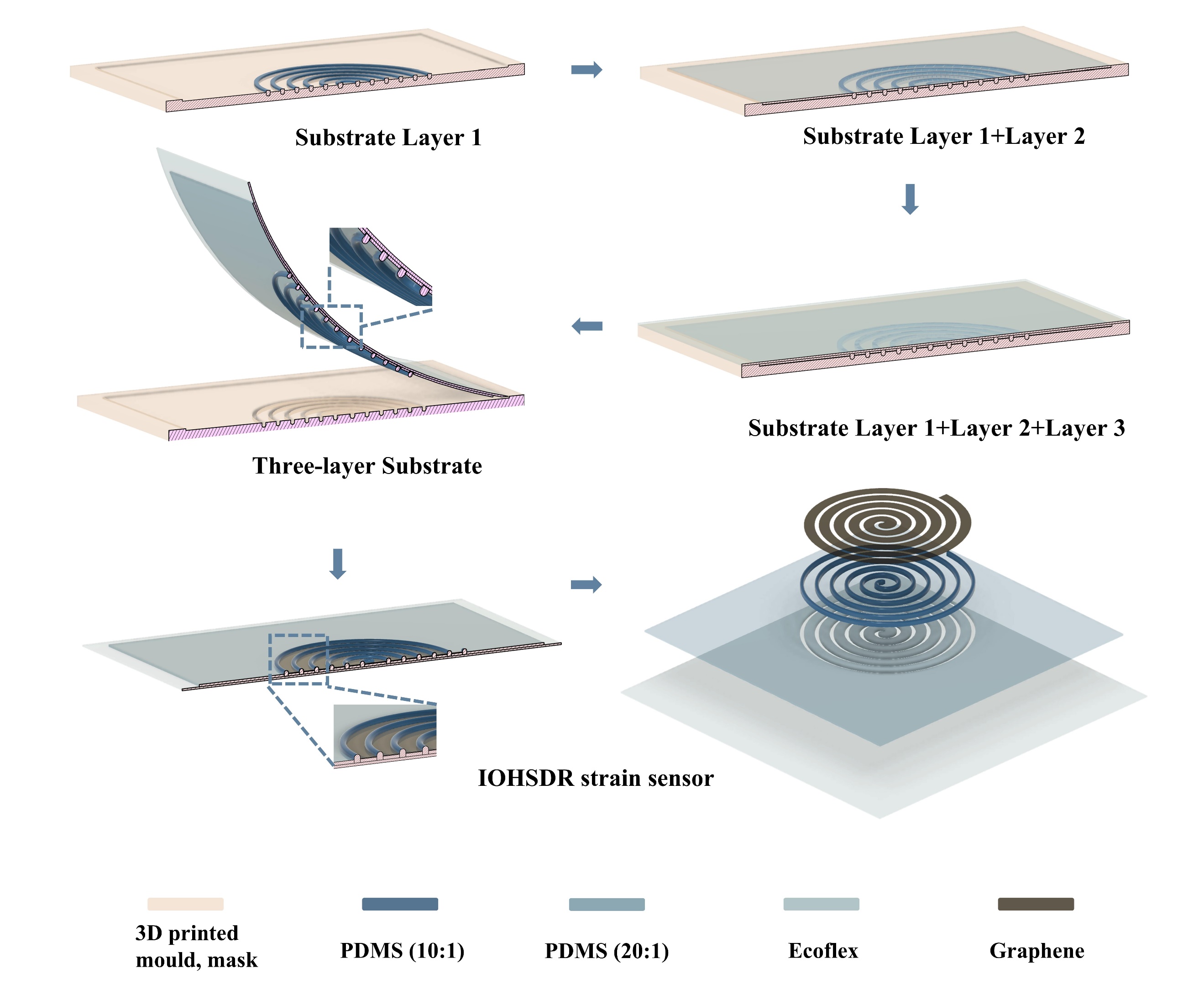
**

**Figure S2.** Sectional view of the fabrication of the IOHSDR strain sensor.


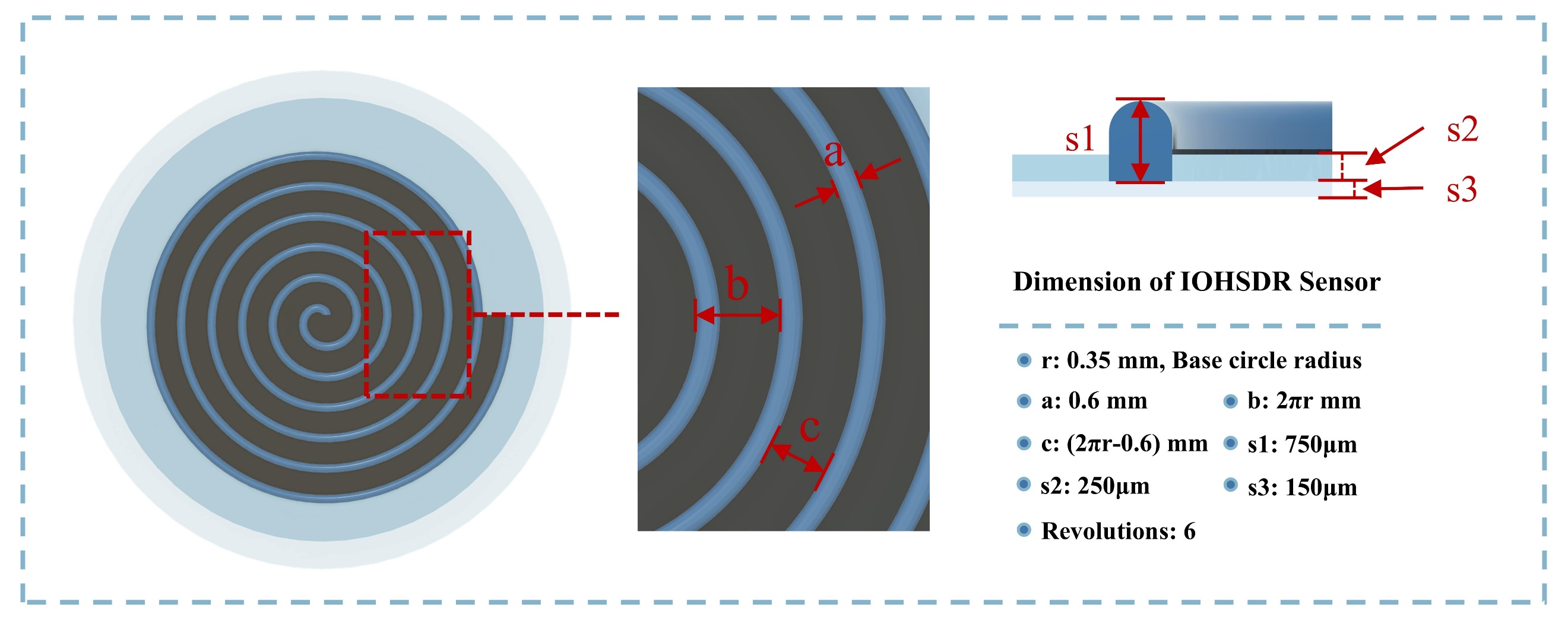


**Figure S3.** Dimension of the IOHSDR strain sensor.

**Figure S4.** Tensile force-strain curve of the IOHSDR device.


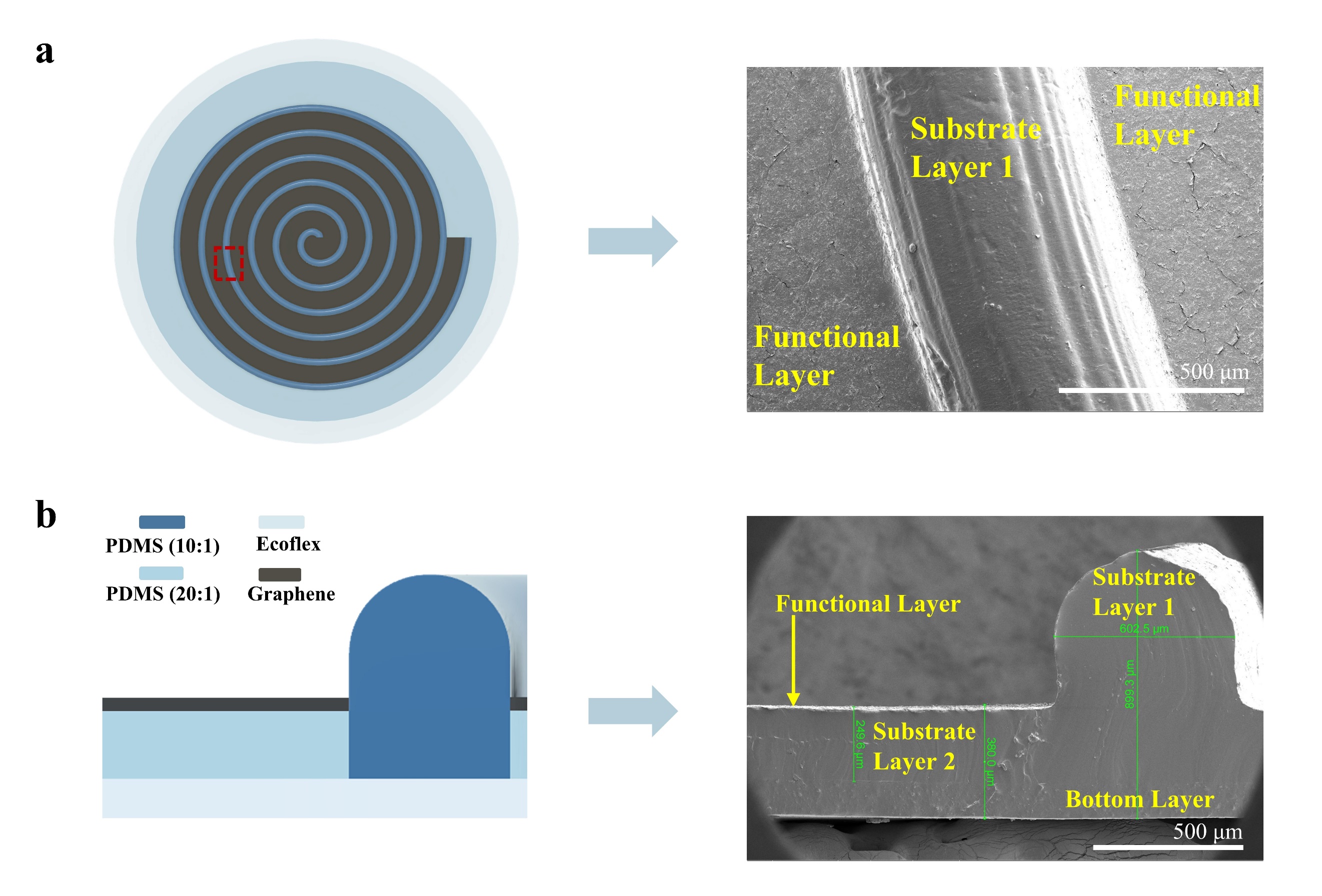


**Figure S5.** SEM images of the IOHSDR device. a) Top view. b) Cross-sectional view.

**
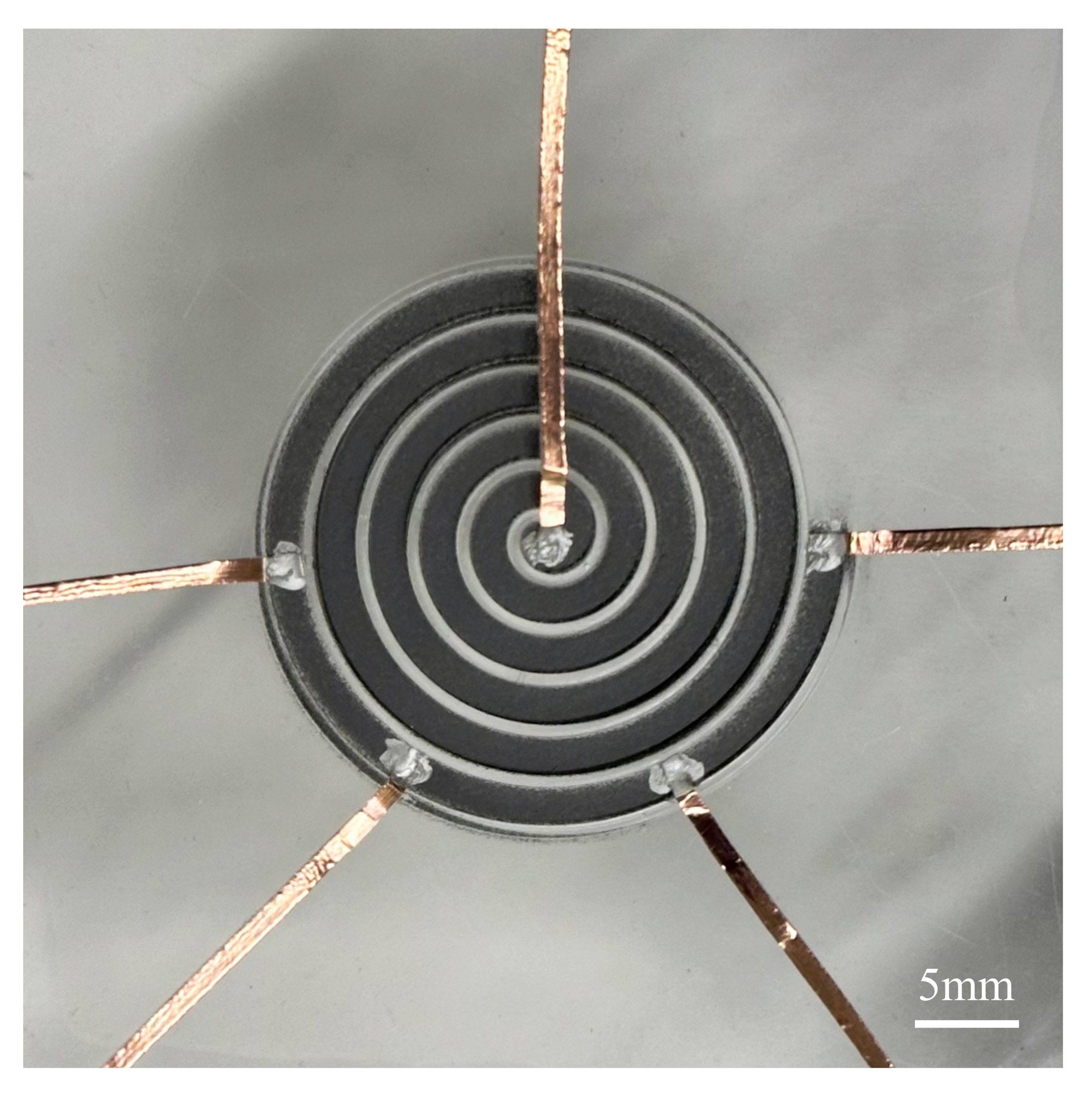
**

**Figure S6.** Photograph of the physical IOHSDR strain sensor.

**Figure S7.** Performance of the IOHSDR strain sensor over a temperature range of 35–45 °C, compared to the throat vibration signal of the word “Cambridge”.


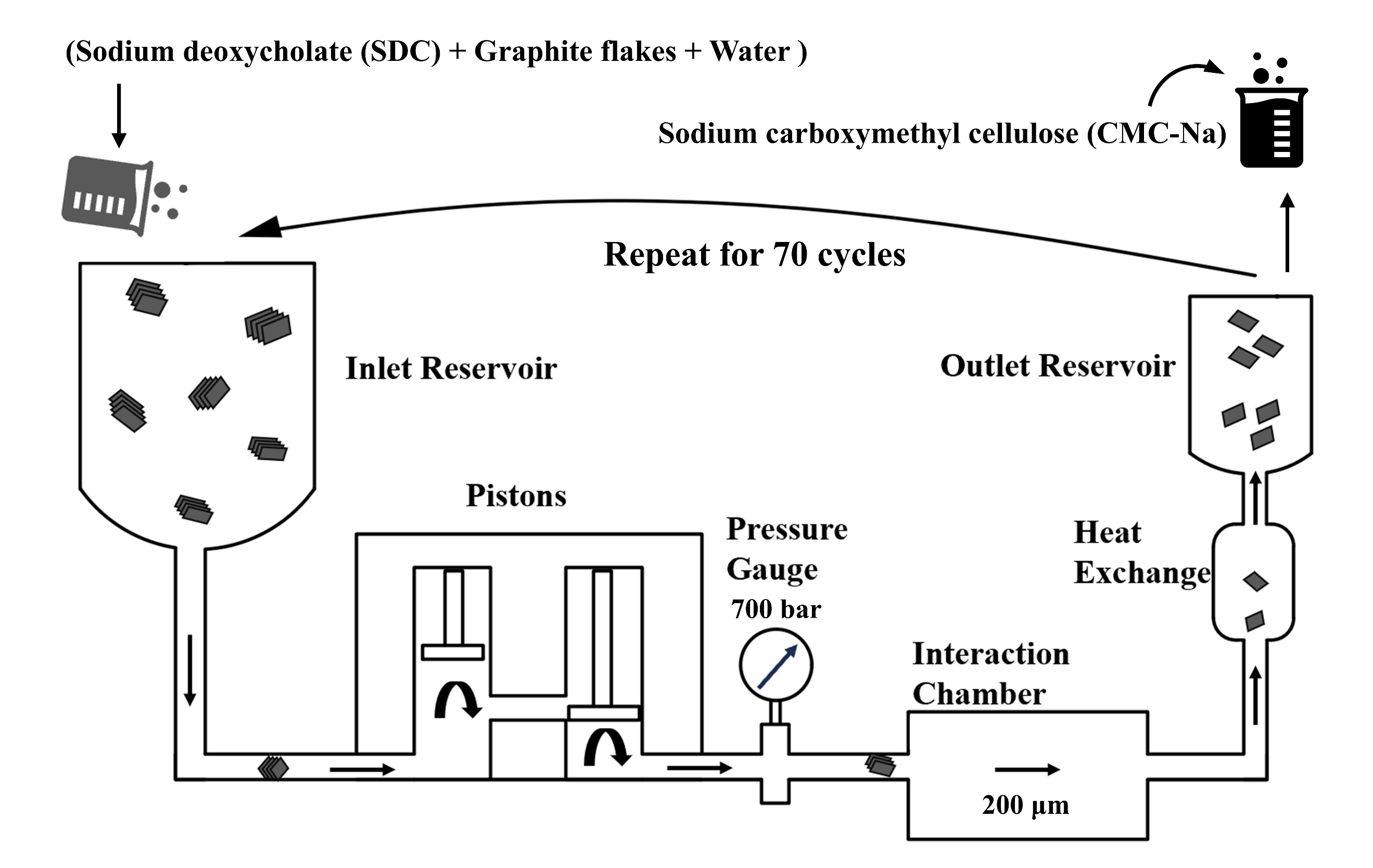


**Figure S8.** Schematic of the functional graphene nanoplatelets ink preparation via High Pressure Homogenizer (HPH).


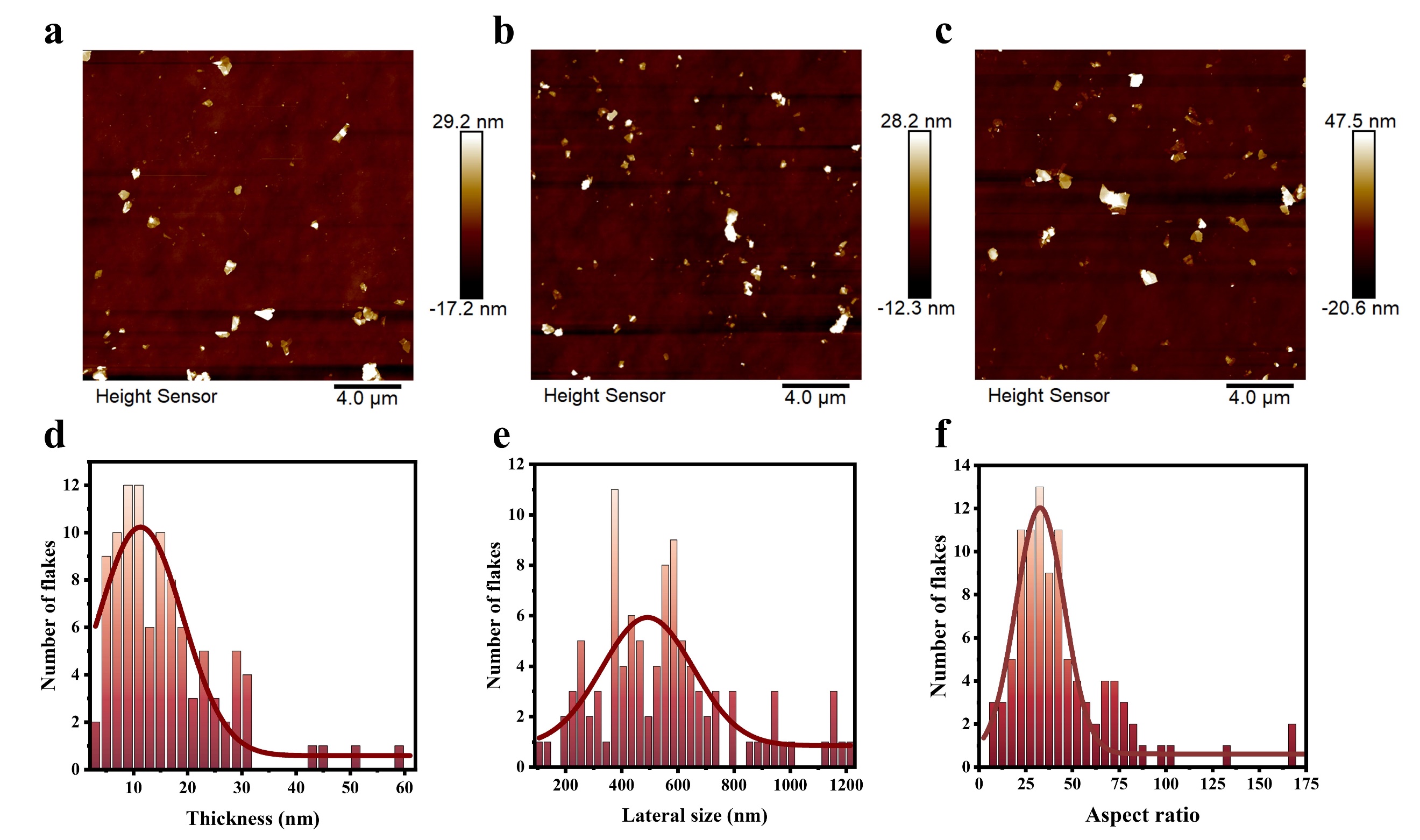


**Figure S9.** Characterization of graphene flakes fabricated by high-pressure homogenizer. a-c) AFM images (20 μm × 20 μm) of graphene flakes scanned at three distinct locations. d-f) Lateral size, thickness, and aspect ratio distributions of graphene flakes, derived from a sample of 100 randomly selected flakes across a-c three AFM scans.


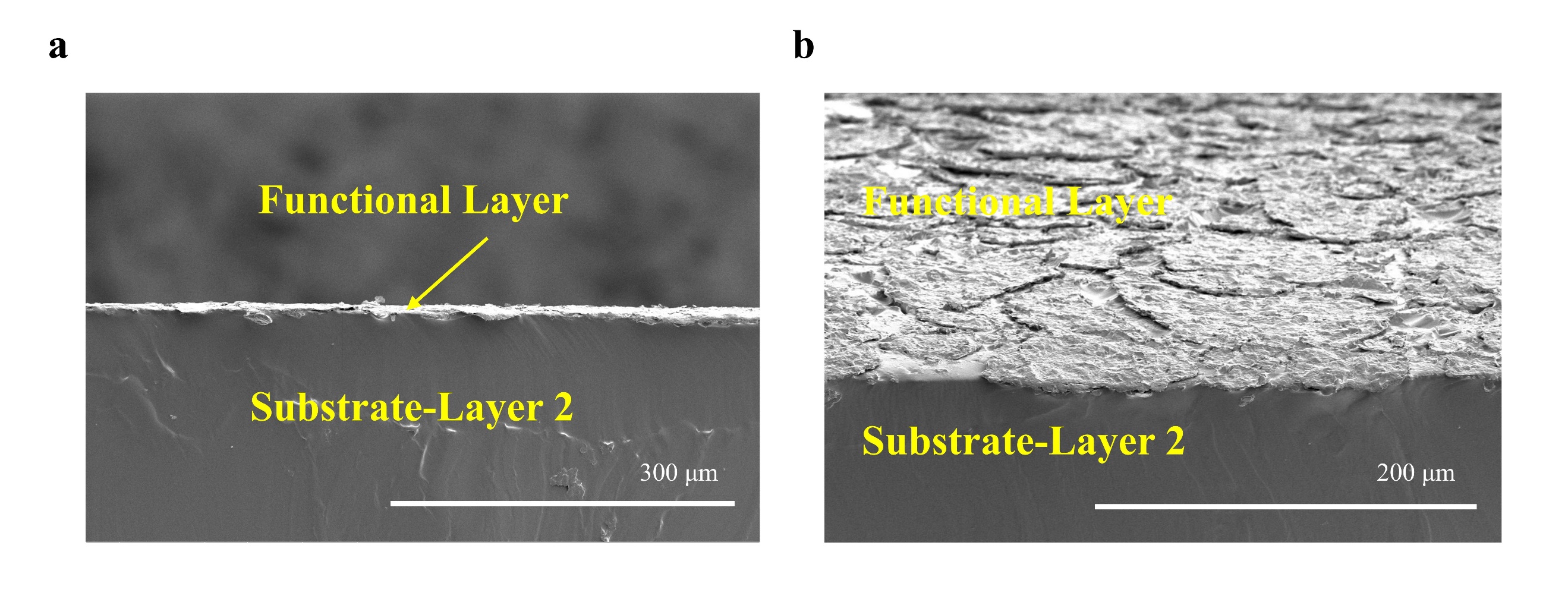


**Figure S10.** Cross-sectional SEM images showing the interface between the functional graphene layer and the substrate layer. a) Without stretching. b) Under stretching.


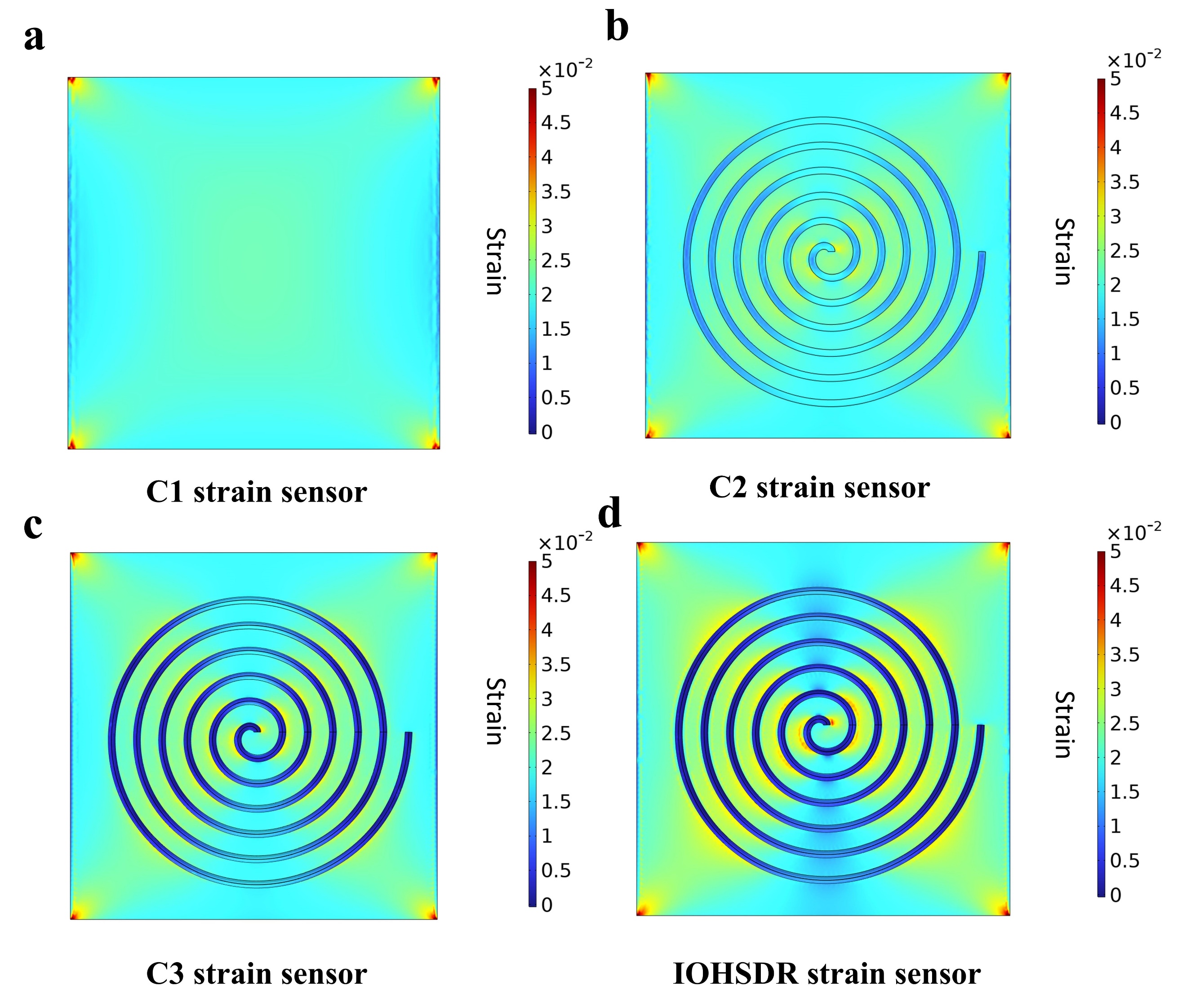


**Figure S11.** Finite element analysis (FEA) simulations of C1, C2, C3, and IOHSDR strain sensors under the 2% strain. The IOHSDR strain sensor shows the largest local strain compared to sensors C1 through C3, following the order: IOHSDR >C3 > C2 > C1. The simulation details are illustrated in Supplementary Note 3.


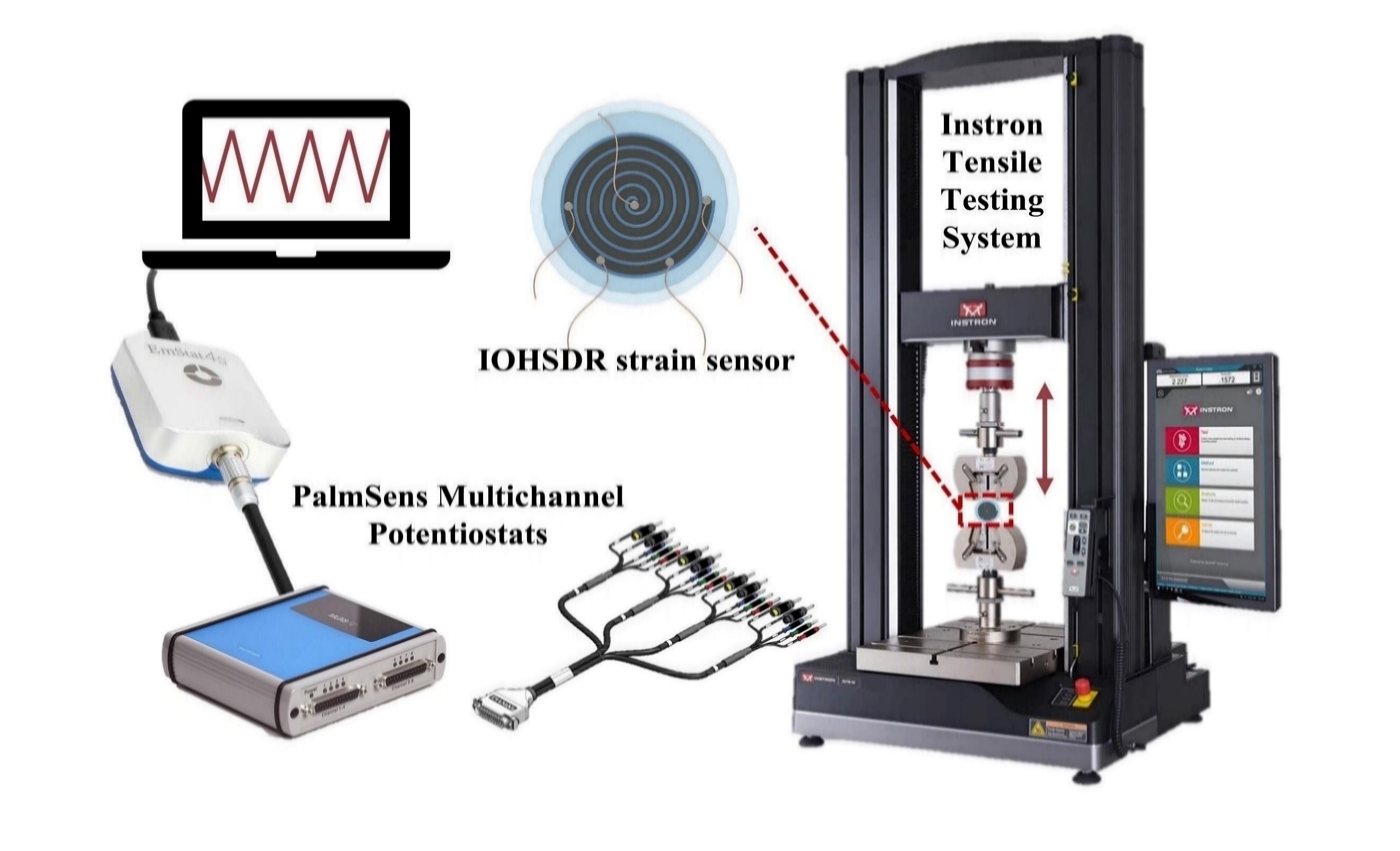


**Figure S12.** Schematic of the strain testing setup, featuring a Multichannel Potentiostats from PalmSens and a tensile testing system from INSTRON. The two ends of potentiostats are connected to a computer and the tensile testing system, respectively. During cyclic stretching applied by the INSTRON system, the four-channel signals from the IOHSDR strain sensor are collected by the potentiostats.

**
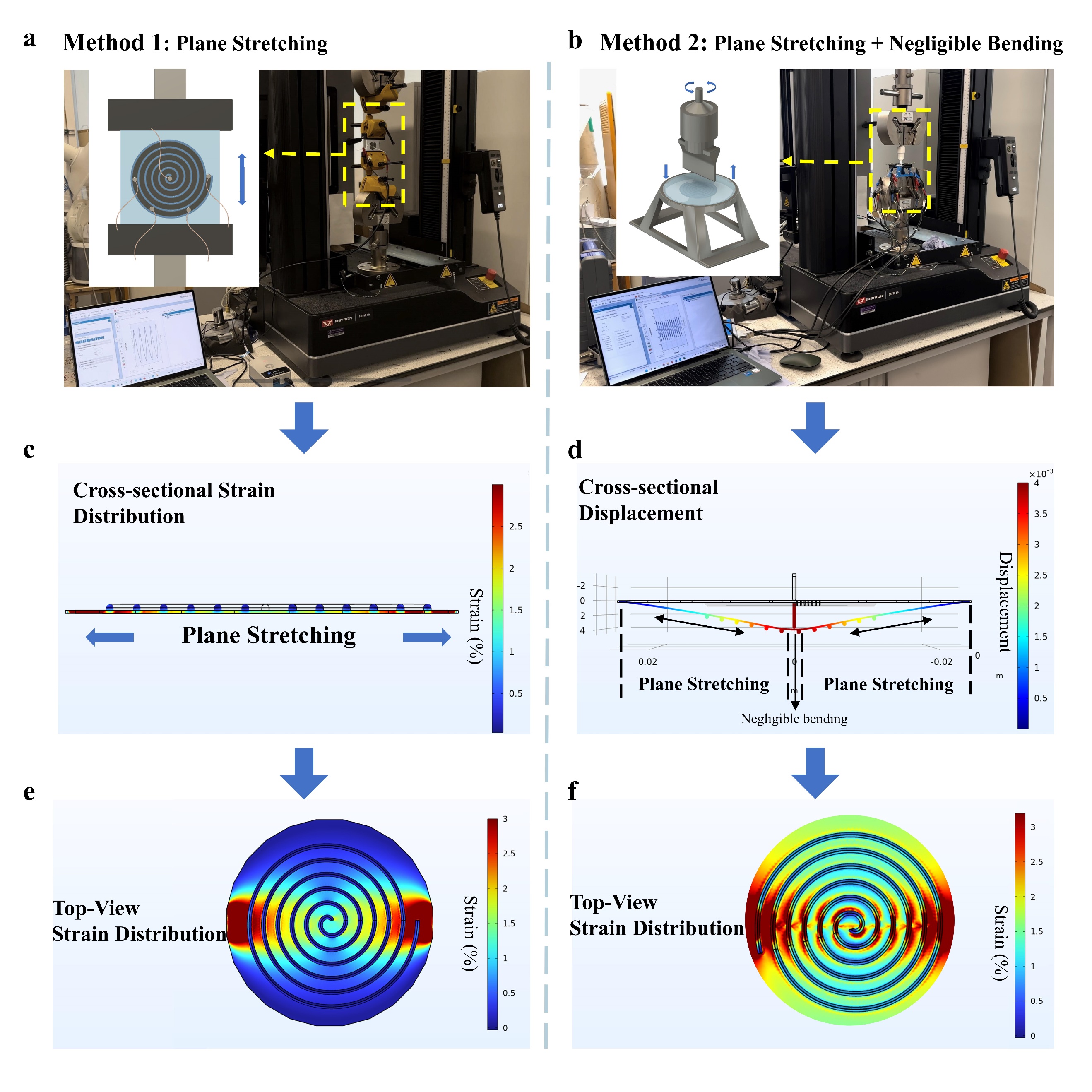
Figure S13.** Two Strain Testing Methods. a) Method 1: Plane stretching. b) Method 2: Plane stretching + negligible bending. c) FEA result of cross-sectional strain distribution when Method 1 is applied. d) FEA result of cross-sectional displacement distribution when Method 2 is applied. e-f) FEA results of top view strain distributions when Method 1 and 2 were applied, respectively.


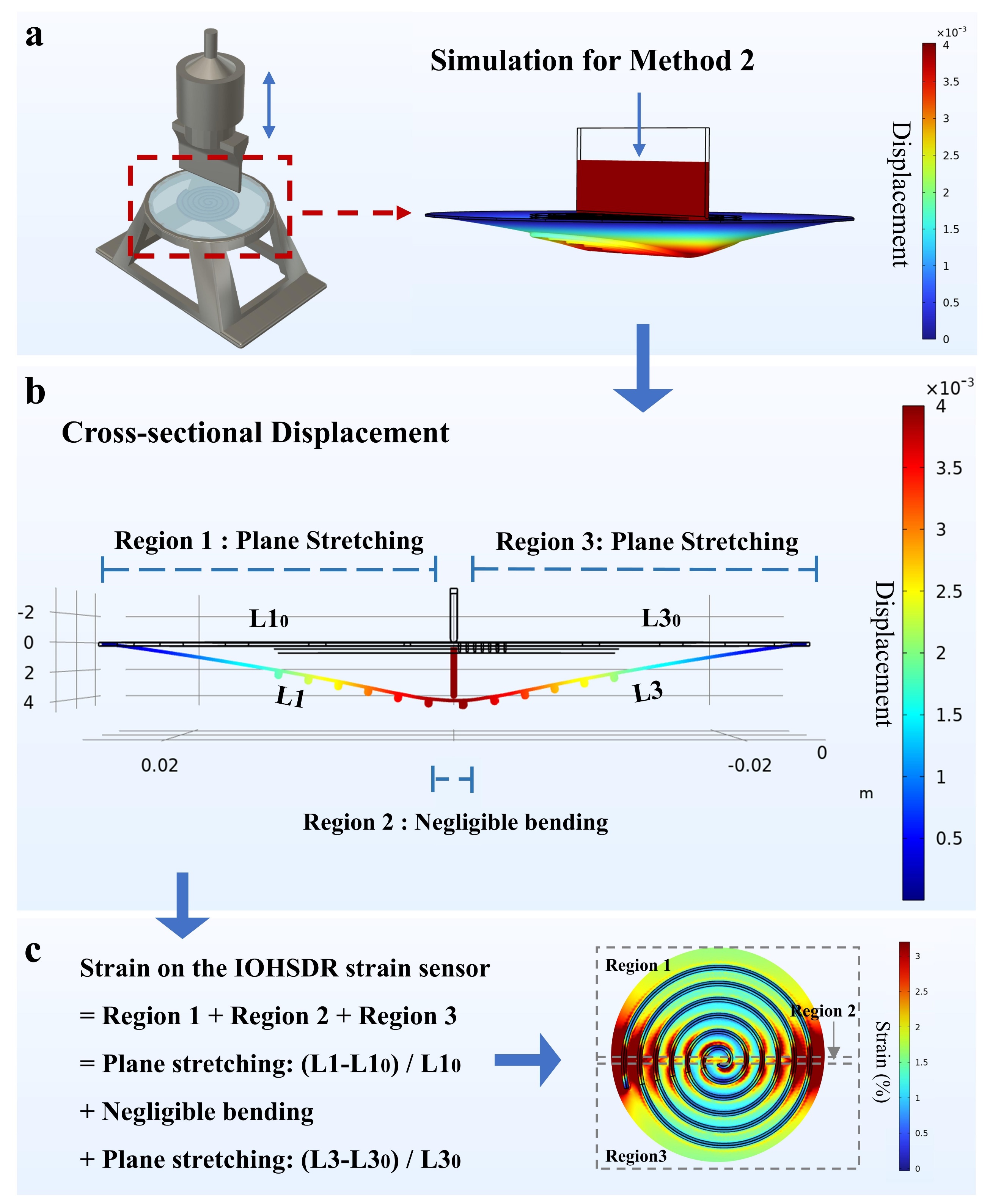


**Figure S14.** Working mechanism of strain testing Method 2. a) Simulation result when Method 2 is applied on the IOHSDR device. b) FEA result of cross-sectional displacement distribution when Method 1 is applied. c) FEA result of top view strain distribution when Method 1 is applied.


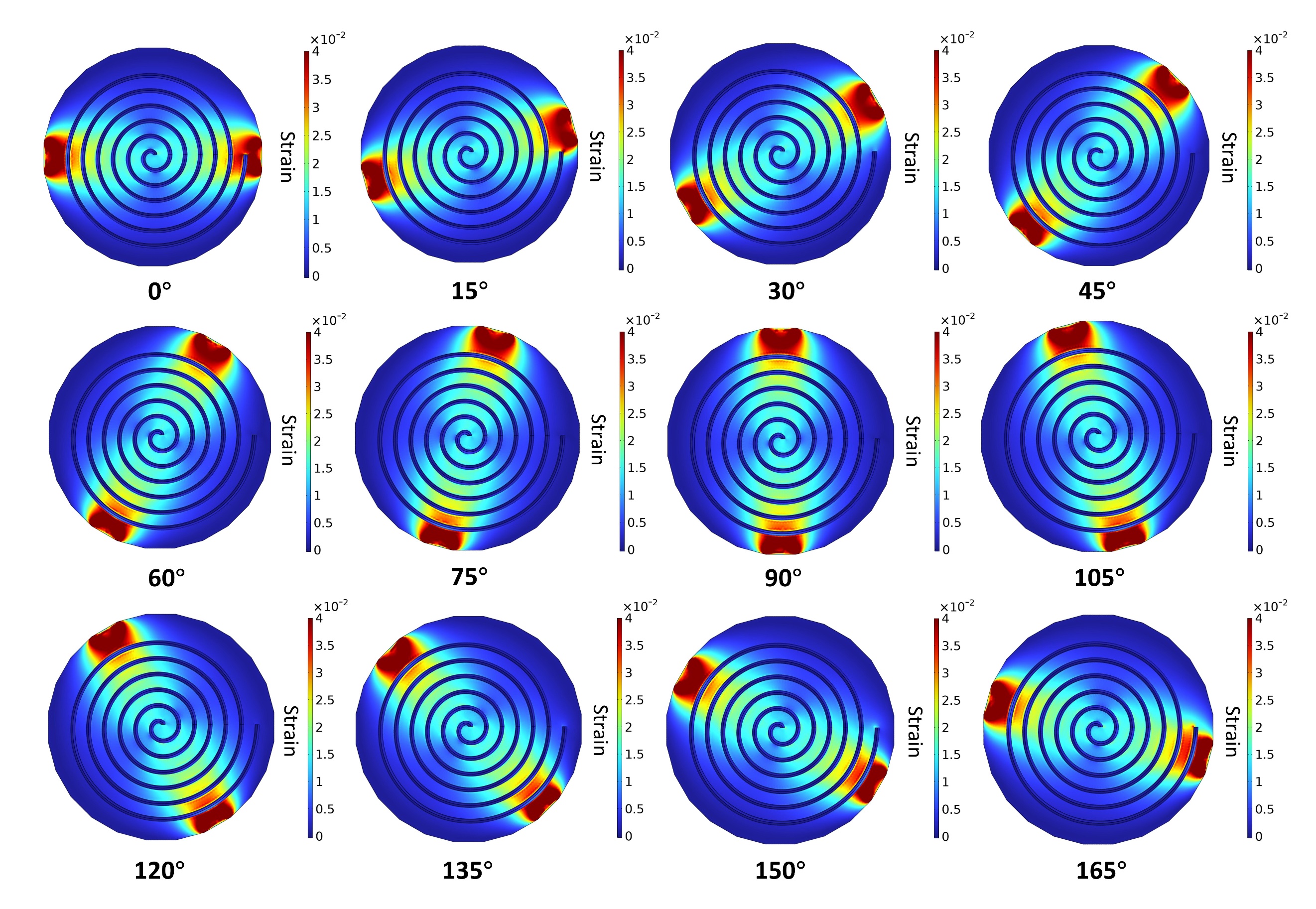


**Figure S15.** Finite element analysis (FEA) simulations of the IOHSDR strain sensor under 2% strain across 12 directions. The results demonstrate a similar strain distribution under stretching from omni directions, highlighting the isotropic omnidirectional strain sensing capability of the IOHSDR strain sensor.


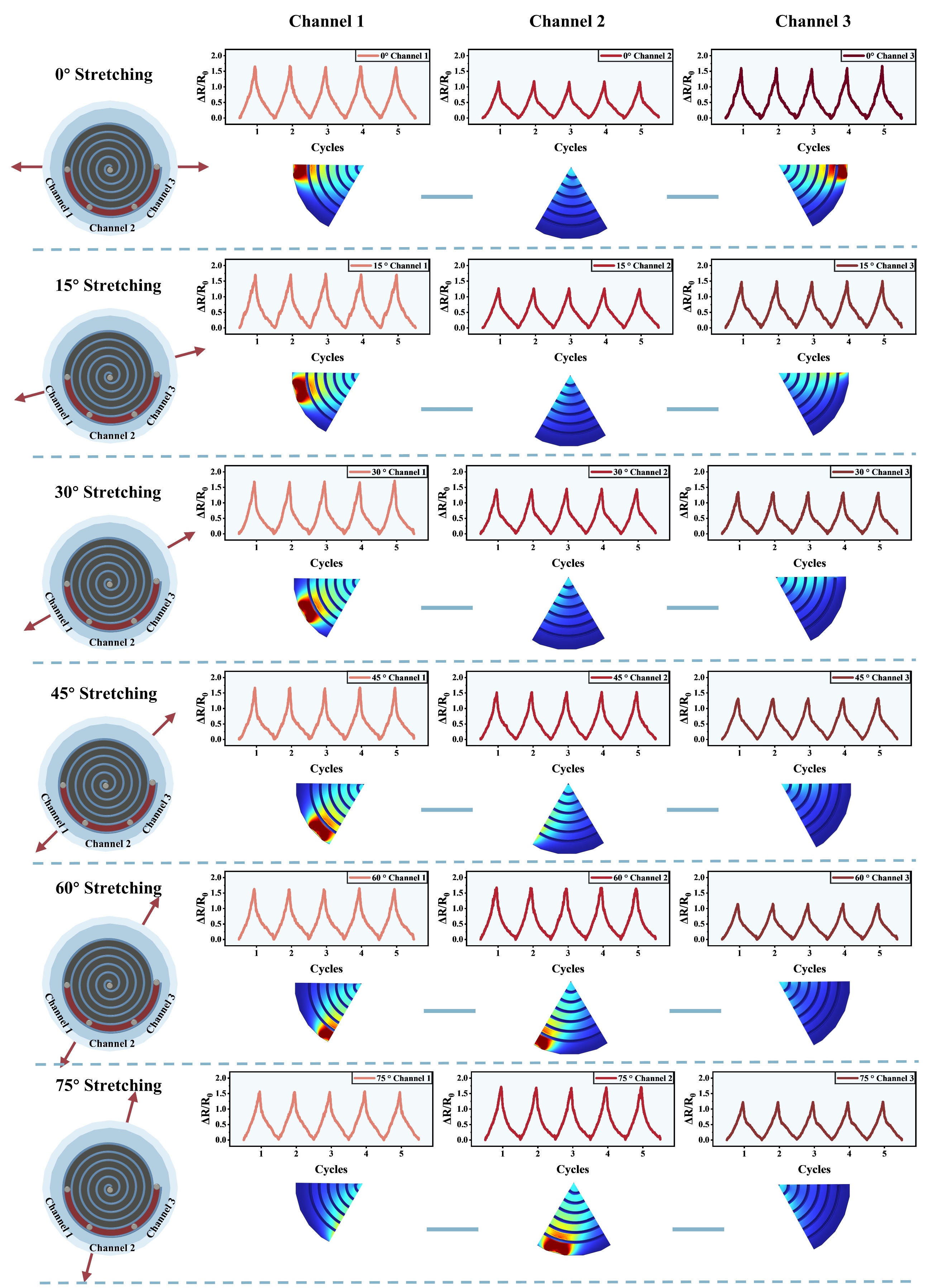


**Figure S16.** Comparison of the three-channel signals for stretching ranging from 0° to 75° at 15° intervals, based on data collected from the IOHSDR strain sensor and finite element analysis (FEA) simulations.


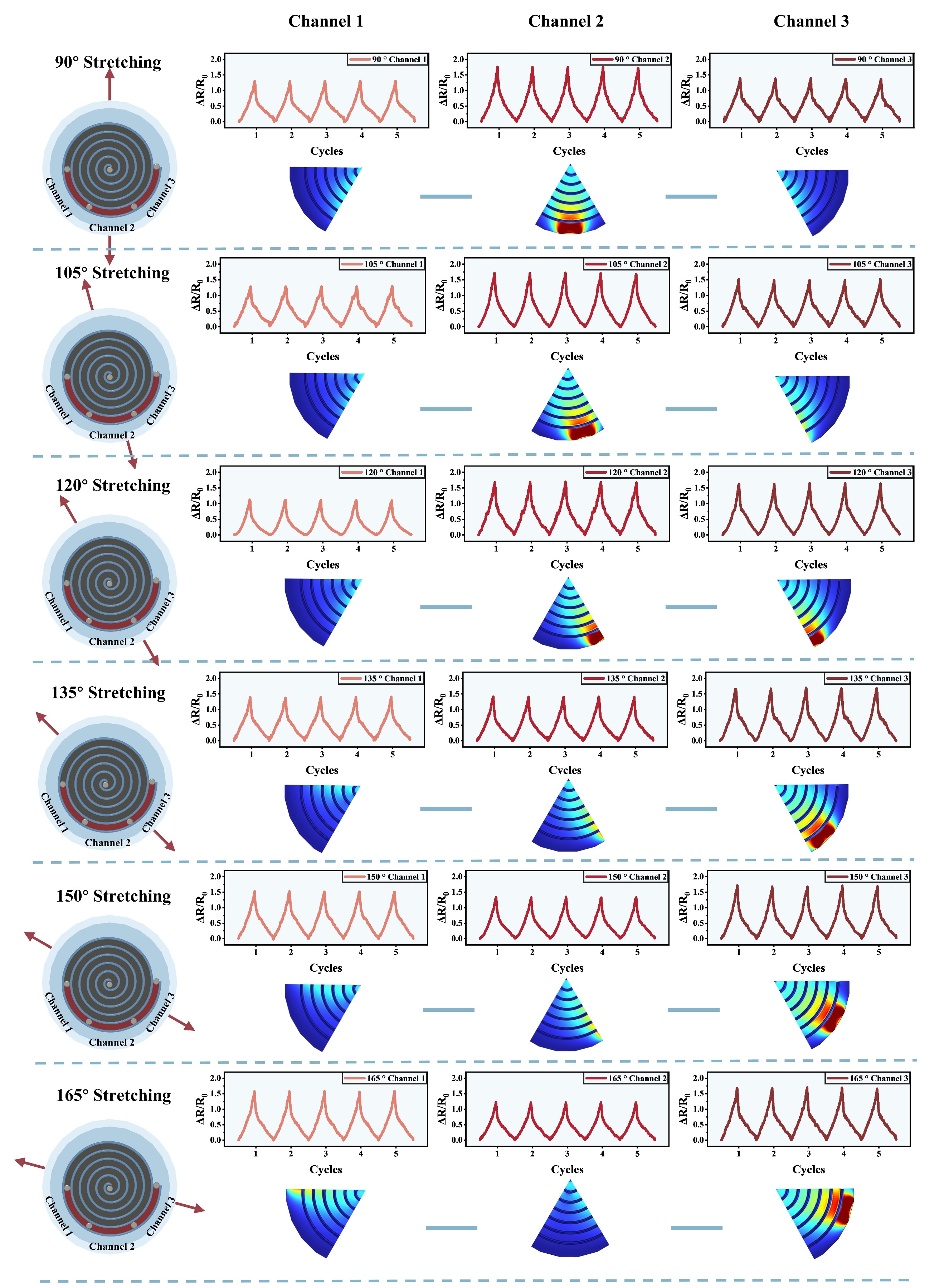


**Figure S17.** Comparison of the three-channel signals for stretching ranging from 90° to 165° at 15° intervals, based on data collected from the IOHSDR strain sensor and finite element analysis (FEA) simulations.


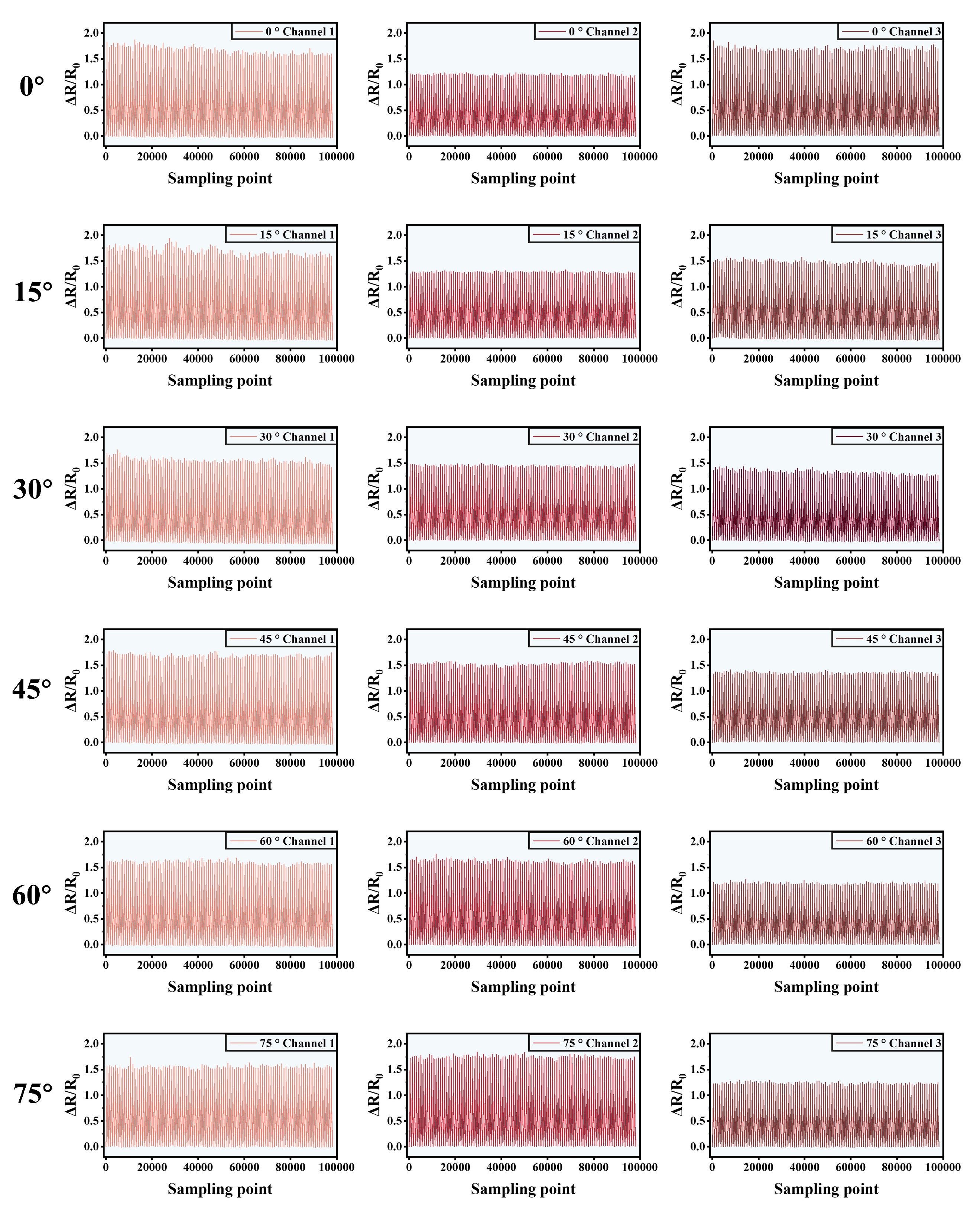


**Figure S18.** A total of 1800 direction-specific resistance data sets (ranging from 0° to 75° at 15° intervals) were collected across three channels of the IOHSDR strain sensor, with each direction consisting of 100 samples per channel.


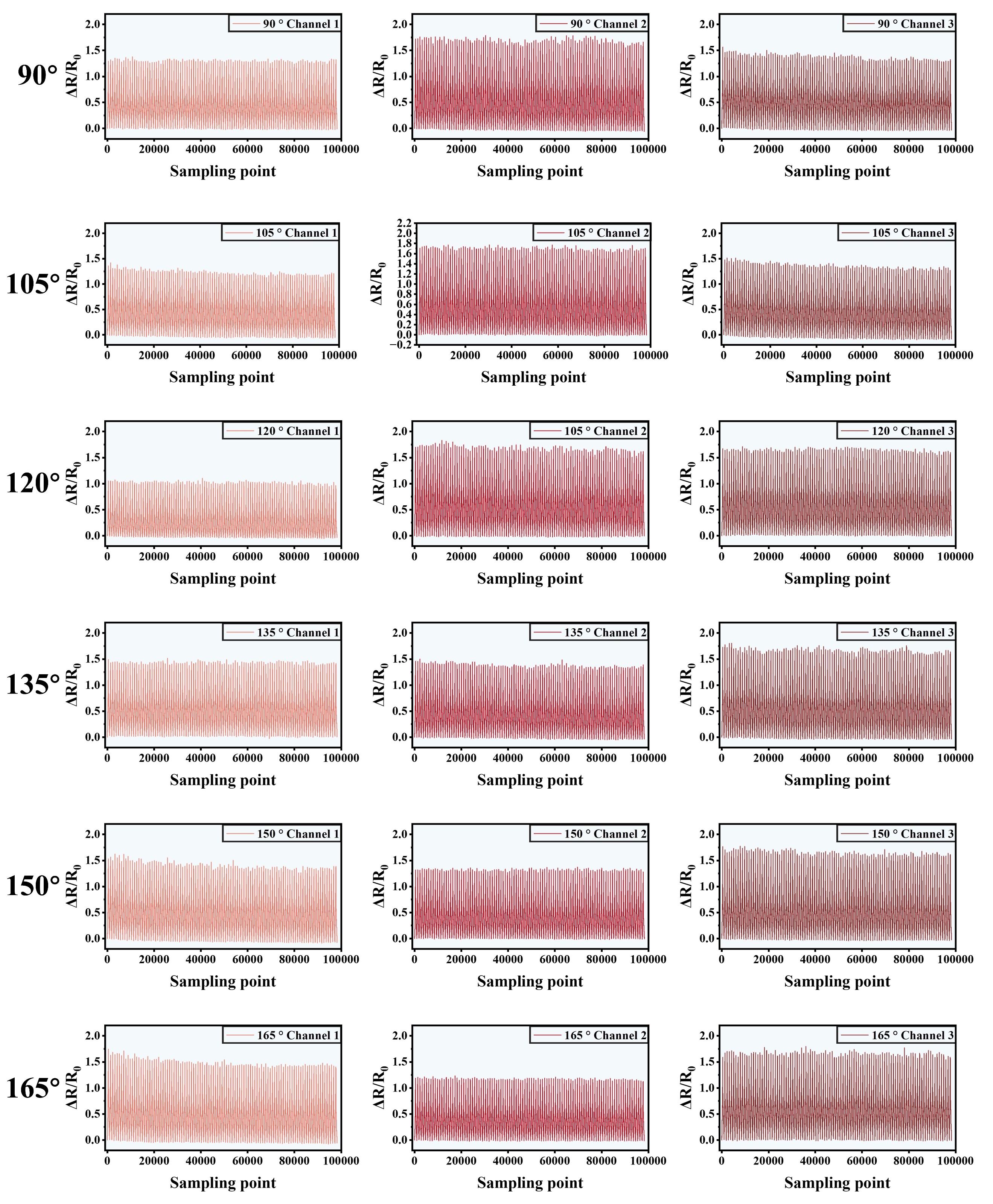


**Figure S19.** A total of 1800 direction-specific resistance data sets (ranging from 90° to 165° at 15° intervals) were collected across three channels of the IOHSDR strain sensor, with each direction consisting of 100 samples per channel.

**Figure S20.** Train and validation loss curves of the proposed model in strain direction recognition. During the training process, we use the cross entropy loss to constrain our model for strain direction recognition. As shown in Figure S9, Supporting Information, the train and validation loss curve both decline promptly and reach convergence within tens of epochs, which demonstrates the stability and efficiency of our model.

**Supplementary Table**

**Table ST1.** Benchmark analysis with other work reported on strain sensors with directionality recognition.

**References**

[1] C. Tang, M. Xu, W. Yi, Z. Zhang, E. Occhipinti, C. Dong, D. Ravenscroft, S.-M. Jung, S. Lee, S. Gao, J. M. Kim, L. G. Occhipinti, *npj Flexible Electron.* **2024**, *8*, 1.

[2] M. P. Wolf, G. B. Salieb-Beugelaar, P. Hunziker, *Prog. Polym. Sci.* **2018**, *83*, 97.

[3] B. Ruben, M. Elisa, L. Leandro, M. Victor, G. Gloria, S. Marina, S. Mian K, R. Pandiyan, L. Nadhira, *Micro Nano Lett.* **2017**, *12*, 754.

[4] A. E. Forte, P. Z. Hanakata, L. Jin, E. Zari, A. Zareei, M. C. Fernandes, L. Sumner, J. Alvarez, K. Bertoldi, *Adv. Funct. Mater.* **2022**, *32*, 2111610.

[5] Y. Yu, D. Sanchez, N. Lu, *J. Mater. Res.* **2015**, *30*, 2702.

[6] A. Borók, K. Laboda, A. Bonyár, *Biosensors* **2021**, *11*, 292.

[7] Y.-S. Yu, Y.-P. Zhao, *J. Colloid Interface Sci.* **2009**, *332*, 467.

[8] O. Akogwu, D. Kwabi, S. Midturi, M. Eleruja, B. Babatope, W. O. Soboyejo, *Mater. Sci. Eng. B* **2010**, *170*, 32.

[9] A. Sharfeddin, A. A. Volinsky, G. Mohan, N. D. Gallant, *J. Appl. Polym. Sci.* **2015**, *132*, app.42680.

[10] J. H. Lee, S. H. Kim, J. S. Heo, J. Y. Kwak, C. W. Park, I. Kim, M. Lee, H.-H. Park, Y.-H. Kim, S. J. Lee, S. K. Park, *Adv. Mater.* **2023**, *35*, 2208184.

[11] J.-H. Lee, J. Kim, D. Liu, F. Guo, X. Shen, Q. Zheng, S. Jeon, J.-K. Kim, *Adv. Funct. Mater.* **2019**, *29*, 1901623.

[12] S.-H. Bae, Y. Lee, B. K. Sharma, H.-J. Lee, J.-H. Kim, J.-H. Ahn, *Carbon* **2013**, *51*, 236.
